# Isolating neural correlates of conscious perception from neural correlates of reporting one's perception

**DOI:** 10.3389/fpsyg.2014.01078

**Published:** 2014-10-08

**Authors:** Michael A. Pitts, Stephen Metzler, Steven A. Hillyard

**Affiliations:** ^1^Department of Psychology, Reed CollegePortland, OR, USA; ^2^Department of Neurosciences, University of CaliforniaSan Diego, La Jolla, CA, USA

**Keywords:** attention, awareness, masking, task-relevance, VAN, P3b

## Abstract

To isolate neural correlates of conscious perception (NCCs), a standard approach has been to contrast neural activity elicited by identical stimuli of which subjects are aware vs. unaware. Because conscious experience is private, determining whether a stimulus was consciously perceived requires subjective report: e.g., button-presses indicating detection, visibility ratings, verbal reports, etc. This reporting requirement introduces a methodological confound when attempting to isolate NCCs: The neural processes responsible for accessing and reporting one's percept are difficult to distinguish from those underlying the conscious percept itself. Here, we review recent attempts to circumvent this issue via a modified inattentional blindness paradigm (Pitts et al., 2012) and present new data from a backward masking experiment in which task-relevance and visual awareness were manipulated in a 2 × 2 crossed design. In agreement with our previous inattentional blindness results, stimuli that were consciously perceived yet not immediately accessed for report (aware, task-irrelevant condition) elicited a mid-latency posterior ERP negativity (~200–240 ms), while stimuli that were accessed for report (aware, task-relevant condition) elicited additional components including a robust P3b (~380–480 ms) subsequent to the mid-latency negativity. Overall, these results suggest that some of the NCCs identified in previous studies may be more closely linked with accessing and maintaining perceptual information for reporting purposes than with encoding the conscious percept itself. An open question is whether the remaining NCC candidate (the ERP negativity at 200–240 ms) reflects visual awareness or object-based attention.

## Introduction

Determining the neural basis of consciousness is one of the most challenging problems in modern cognitive neuroscience. Much progress has been made over the past 25 years by simplifying the problem and focusing first on identifying neural correlates of conscious perception or “NCCs” (Baars, 1989; Logothetis and Schall, 1989; Crick and Koch, 1990, 2003). The primary strategy has been to compare brain activity elicited by physically identical stimuli of which subjects are aware vs. unaware. While appealing in its simplicity, this approach has recently been criticized for being too inclusive in what counts as an NCC (Aru et al., 2012; de Graaf et al., 2012). Depending on how awareness is manipulated, neural mechanisms that are necessary-but-not-sufficient or sufficient-but-not-necessary for conscious perception have often been misinterpreted as true-NCCs. Aru et al. (2012) refer to such neural processes as “pre-requisites” and “consequences” of conscious perception, respectively, and have encouraged researchers to develop new paradigms to help distinguish the “NCC-proper” from these related, yet functionally distinct, processes.

### Terminology and theoretical considerations

While the central tenets of Aru et al.'s (2012) proposal are well-justified, we prefer to use the terms “pre-conscious” and “post-perceptual” instead of “pre-requisite” and “consequence.” In our view, the term pre-requisite is too general and the term consequence too restrictive. For example, retinal processing could be considered a pre-requisite of visual awareness (with the exception of TMS-induced phosphenes); however, no one has proposed retinal activity as a potential NCC. The term *pre-conscious*, instead, is used here to refer only to cortical and cortico-thalamic activity following the initial feedforward activation of primary sensory cortex. Pre-conscious processing immediately precedes (both temporally and functionally) conscious processing and is capable of establishing elaborate perceptual representations that may become conscious (Dehaene and Naccache, 2001; Dehaene et al., 2006). The term consequence, on the other hand, appears to be used by Aru and colleagues to refer to certain neural events that *necessarily* follow the conscious awareness of a stimulus. In contrast, the term *post-perceptual* leaves open the possibility that conscious perception may occur with or without these subsequent processing events. As outlined below, simple manipulations of the task can eliminate post-perceptual processing while leaving conscious perception intact.

It should also be noted that the term “perceptual awareness” (often abbreviated as “awareness”) will be used here to describe situations in which perceptual content is reportable, although actual reporting may not take place, e.g., because the subject has been instructed to only report awareness of certain types of stimuli. This reportability requirement is commonly used to operationally define conscious perception but differs from certain theoretical frameworks that consider some types of perceptual processing as “phenomenally conscious” even if the subject is unable to report anything about these percepts when specifically instructed to do so (Lamme, 2006; Vandenbroucke et al., 2014). It also differs from notions of phenomenal consciousness in which perceptual content is accessible, yet not necessarily accessed (Block, 2007, 2011). In both cases, we consider such processing as “pre-conscious” rather than a special type of unreportable consciousness, and our main goal is to isolate neural correlates of “access consciousness” from correlates of pre-conscious and post-perceptual processing (Dehaene and Changeux, 2004, 2011; Block, 2005; Dehaene et al., 2006). Because the term “access” can have different meanings in different contexts, we will qualify its usage for clarity, e.g., “conscious access” typically refers to global availability of perceptual information for flexible use by a variety of cognitive systems, while “access of perceptual information for report” refers to a much narrower set of post-perceptual operations involved in task-related memory and decision-making processes.

While it is important to separate both pre-conscious and post-perceptual activity from correlates of conscious perception, the current study focuses mainly on post-perceptual processing. To illustrate the problem of conflating neural correlates of post-perceptual processing with correlates of conscious perception, it is useful to consider “the refrigerator door problem” (a neural counterpart to the “refrigerator light illusion” described by Block, 2001). Imagine you have no access to the internal machinery of a refrigerator and your goal is to determine under what conditions the light inside the refrigerator turns on. You might start by opening the door to check and proceed to try just opening it a crack or just for a brief moment. However, every time you open the door the light is always on and you can't be sure the light would have been on if you had kept the door closed. In consciousness research, one of the goals is to determine whether unique brain signal X correlates with conscious perception (the light turning on), but to do so requires a perceptual report from the subject (opening the door). The problem is that it is often difficult to determine whether the same brain signal X would have occurred if the subject had not accessed this information for report (kept the door closed).

### Post-perceptual processing in paradigms that manipulate visual awareness

To manipulate visual awareness in the lab, the most commonly used paradigms include backward masking, the attentional blink, change blindness, binocular rivalry, and signal detection at threshold (e.g., Pins and ffytche, 2003; Fernandez-Duque et al., 2003; Sergent et al., 2005; Koivisto et al., 2006; Pitts et al., 2010). In all cases, subjects are asked to provide some type of perceptual report after each trial. These reports range from identification of target stimuli to detailed visibility ratings such as using a perceptual awareness scale (Ramsøy and Overgaard, 2004). At first glance, this method of requiring a subjective report after each trial seems unproblematic. Regardless of whether the stimulus was consciously perceived, the subject always has to report something even if the report is “I saw nothing.” Indeed, in many paradigms, stimuli are visible on roughly half of the trials, and there would be no means of sorting trials into aware vs. unaware conditions without trial-by-trial reports. However, upon closer scrutiny, aware vs. unaware contrasts in paradigms such as these are likely to expose not only differences in brain activity related to conscious perception but also differences in post-perceptual processing. On aware trials, subjective reports rely on the maintenance of perceptual information in working memory and access of this information by higher-level cognitive systems that enable decision-making and response planning/execution. On unaware trials, there is no conscious perceptual information to maintain or access even though a decision must be made and a negative response must be planned and executed. Thus, in addition to differences in conscious perception, these two types of trials differ in terms of post-perceptual processing such as maintenance in working memory and access of perceptual information for decision-making.

**Figure 1:**
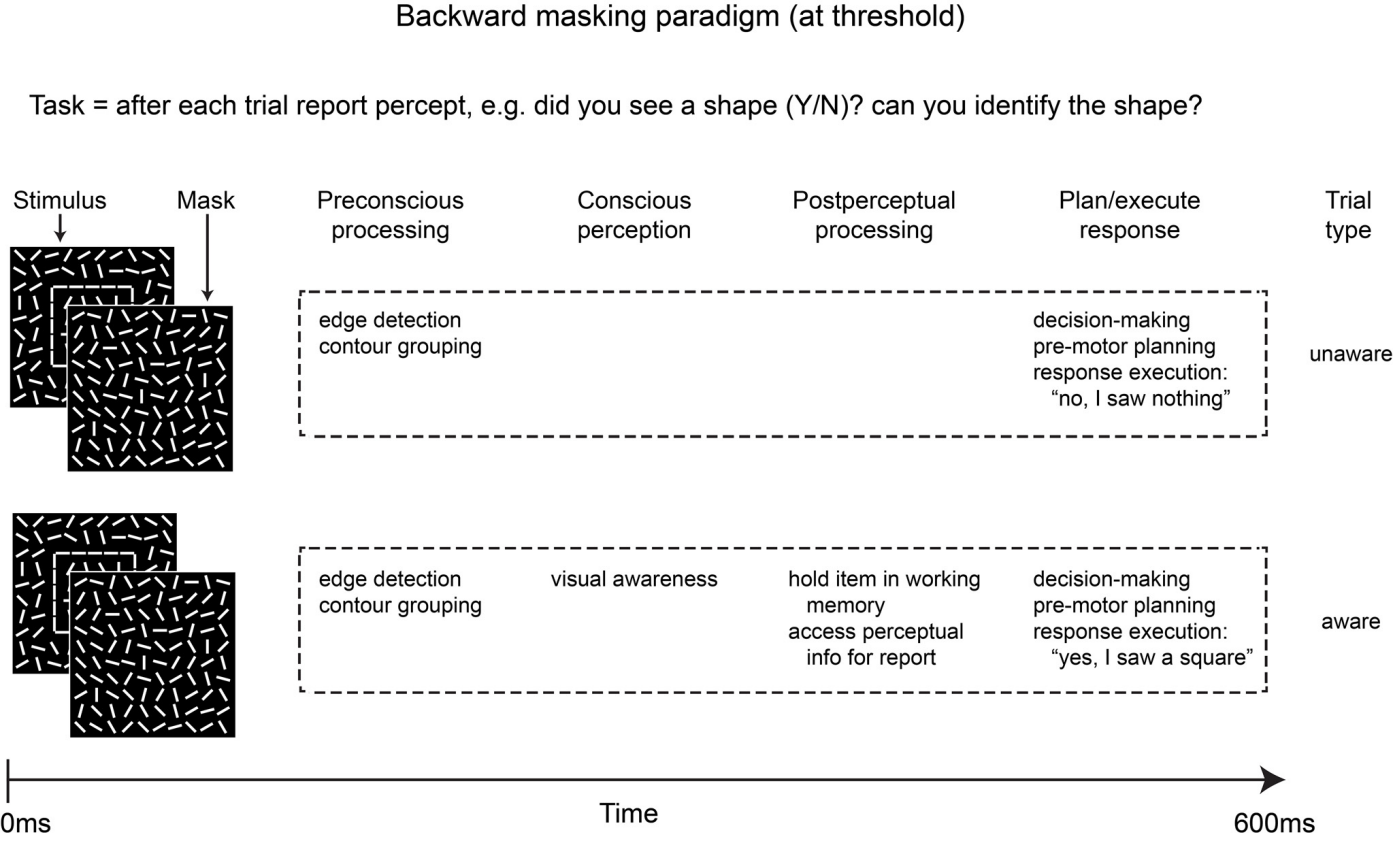
**Simplified schematic depicting some of the stages of processing involved in the two main trial types of a typical backward masking experiment in which the stimulus is masked at a constant SOA (e.g., 50 ms) and is visible on roughly 50% of trials**. In addition to differences in conscious perception, post-perceptual processing is likely to differ across the two trial types due to the task demands.

Figure 1 provides a simplified schematic outline of some of the stages of processing likely to be involved in aware vs. unaware trials for a typical backward masking experiment in which the stimulus (e.g., an outline square) is perceived on roughly half of the trials. Note that in addition to differences in visual awareness, the two trial types also differ in post-perceptual processing, thus any differences in neural activity between aware and unaware trials may reflect post-perceptual maintenance and access for report instead of awareness *per se*. Importantly, the attentional blink, change blindness, signal detection, and a number of other paradigms are vulnerable to this same confound.

### The sustained inattentional blindness paradigm

As an alternative to these more commonly employed paradigms to manipulate awareness, we recently adapted the inattentional blindness paradigm, originally developed and extensively tested in behavioral studies by Mack and Rock (1998), for use in conjunction with electrophysiological recordings of brain activity (Pitts et al., 2012). This paradigm includes three experimental phases. In the first phase, subjects perform a distracter task while a critical (unexpected) stimulus is presented directly in the center of their view. After 200 or more presentations of the critical stimulus, the subjects are queried regarding their awareness of this stimulus. Typically, about half of all subjects report a complete lack of awareness of the critical stimulus and are thus deemed inattentionally blind in this first phase. Importantly, after being asked about the critical stimulus, subjects are instructed to “continue performing the same task as before” in the second phase of the experiment. Due to the intervening questions, all subjects become aware of the critical stimulus during phase 2, but because they are performing the same distracter task they do not need to access information about the critical stimulus for immediate perceptual report. Finally, in the third phase of the experiment, subjects are instructed to forego the distracter task and perform a discrimination task in which the critical stimulus becomes task-relevant.

**Figure 2:**
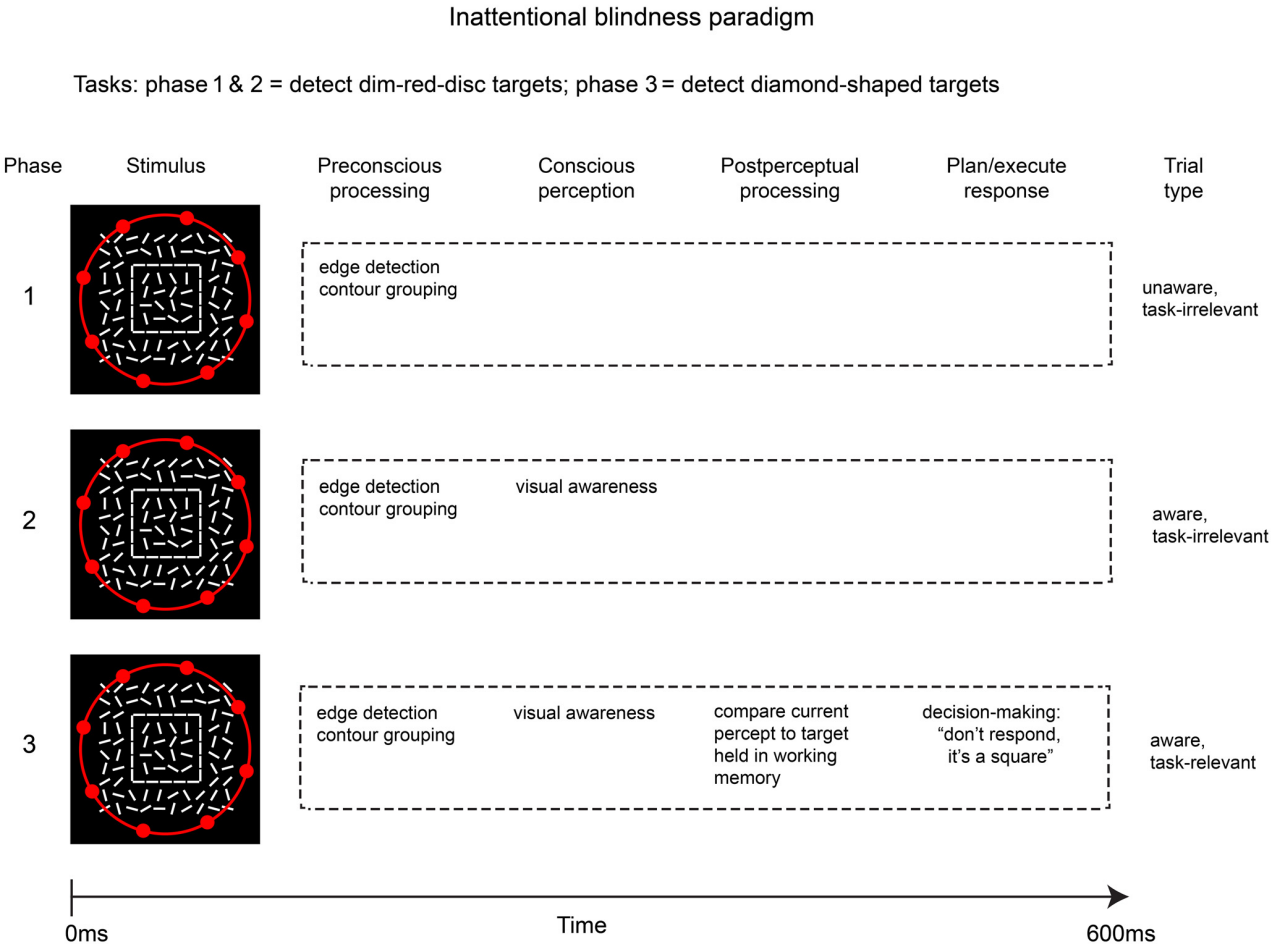
**Outline of the modified inattentional blindness paradigm used by Pitts et al. (2012)**. Because the critical stimulus (square shape) is task-irrelevant in phases 1 & 2, post-perceptual processing is avoided while allowing a contrast that better isolates differences in conscious perception. Neural correlates of post-perceptual processing can be evaluated by comparing phases 3 & 2.

This modified inattentional blindness paradigm allows comparisons of brain activity across three conditions in which awareness and post-perceptual processing are manipulated in a step-wise fashion: phase 1 = unaware; phase 2 = aware without post-perceptual processing (critical stimulus is task-irrelevant); phase 3 = aware with post-perceptual processing (critical stimulus is task-relevant). Figure 2 sketches a simplified overview of the stages of processing involved in the three phases of this inattentional blindness paradigm.

The contrasts of interest in this three-phase experiment are between brain activity elicited by the critical stimulus in phase 2 vs. phase 1 (i.e., awareness vs. unawareness of task-irrelevant stimuli) and phase 3 vs. phase 2 (i.e., task-relevant vs. task-irrelevant stimuli). The first contrast (phase 2 vs. 1) should reveal neural correlates of conscious perception *per se*, while the second contrast (phase 3 vs. 2) isolates correlates of post-perceptual processing (maintenance in working memory and access for report). It should be noted that direct comparisons of brain activity across phases would be of questionable validity because the ordering of the three phases cannot be counterbalanced and neural activity may differ due to repeated exposure to the stimuli, neural fatigue, sensory adaptation, etc. To circumvent this issue, a control stimulus (e.g., a randomized background having no shapes or features) can be randomly intermixed with the critical stimuli in each phase, and neural responses to the critical stimulus can first be contrasted with neural responses to the control stimuli *within* each phase prior to making across-phase comparisons. Also, in order to prevent contamination by pre-motor and motor-related activity, the critical stimuli are never targets that require manual responses, even in the third phase of the experiment. Instead, an infrequent target stimulus is also intermixed in all phases of the experiment, and during the third phase subjects must discriminate between the critical (non-target) stimulus and this target stimulus in order to respond appropriately.

### Pre-conscious, conscious, and post-perceptual EEG signatures in the sustained inattentional blindness paradigm

Using this modified inattentional blindness paradigm, a potential NCC-proper was isolated from pre-conscious and post-perceptual activity (Pitts et al., 2012, 2014; Pitts and Martinez, 2014). Event-related potentials (ERPs) elicited by shape stimuli (formed by oriented line elements) were compared to randomly oriented control stimuli across the three phases of the experiment. During all three phases (including inattentional blindness), an initial occipital negativity was evident from ~160–220 ms. We interpreted this activity as pre-conscious because it was evident even when subjects did not report any conscious awareness of the shape stimuli due to inattentional blindness induced by performing the distracter task. We initially referred to this component as “Nd1” for “negative difference 1” (Pitts et al., 2012), but have subsequently labeled it “CIN” for “contour integration negativity” (Pitts and Martinez, 2014), on the assumption that it reflects pre-conscious neural activity associated with extracting contour information from the stimulus array. In the second and third phases (aware conditions), the CIN was followed by a bilateral occipital-parietal negativity from ~200–300 ms (initially labeled “Nd2”), which was similar in timing and scalp topography to the previously reported “visual awareness negativity” or “VAN” component (Koivisto and Revonsuo, 2010; Railo et al., 2011). Importantly, this negativity was also elicited during phase 1 in the group of subjects who later reported having spontaneously noticed the (irrelevant) shape stimulus. When the shape stimuli were task-irrelevant, no ERP differences were evident subsequent to the VAN, as subjects were not required to further process this information. In the third phase, however, the VAN was followed by a selection negativity (SN) and two late positive components (a late occipital positivity “LOP,” and the centro-parietal P3b) as well as by induced gamma oscillations (Pitts et al., 2012, 2014). We interpreted these latter effects as post-perceptual because they were absent in the second phase (aware, task-irrelevant) and present in the third phase (aware, task-relevant).

In a subsequent experiment (Shafto and Pitts, 2013), we tested a different critical stimulus, line drawings of faces, in a similar inattentional blindness paradigm. Here, we found that the face-specific N170 component as well as the VAN were absent during inattentional blindness (phase 1) but were present in both aware conditions (phase 2 and 3). Consistent with the results from the shape experiment, the SN, LOP, P3b, and induced gamma oscillations were only evident in the third phase in which the face stimuli were task-relevant. Importantly, in both experiments we were able to compare brain activity in aware vs. unaware conditions (phase 2 vs. phase 1-unaware subjects) without requiring immediate access for perceptual report. This was accomplished by presenting the stimuli well above threshold (300 ms duration, easily visible if expected) and by delaying the perceptual report until after the entire block of trials (which lasted ~10 min and delivered 200+ stimuli). In both the shape and face experiments, subjects who were initially inattentionally blind often expressed genuine surprise at having failed to notice such salient stimuli for 10 min, and many subjects reported “not being able to avoid noticing the stimuli” once they knew the stimuli were being presented (in phase 2). These design features are critical in paradigms in which trial-by-trial perceptual reporting is intentionally avoided; i.e., one must be certain that the critical stimuli are never seen during the unaware condition and that these same stimuli are obvious and readily visible during the aware condition, even when subjects are performing a separate task.

This modified inattentional blindness paradigm shows promise in helping to distinguish pre-conscious and post-perceptual activity from the NCC-proper. Future fMRI and intra-cranial EEG studies should consider adopting similar methods. However, one drawback with the inattention paradigm is that it only includes 3 of the possible 4 combinations of visual awareness and task-relevance; i.e., the unaware, task-relevant condition, which is common in other awareness paradigms, is absent in the inattentional blindness paradigm. This missing condition, in which a stimulus is attended and relevant to the task but not consciously perceived, may be important for separating neural correlates of awareness from neural correlates of attention. It is also advantageous to cross-validate results by employing more than one type of experimental paradigm, and the above mentioned results have only been obtained so far using the inattentional blindness approach. The current study was designed to address these outstanding issues.

### A 2 × 2 manipulation of visual awareness and task-relevance in a masking paradigm

One option for measuring NCCs using the masking paradigm is to present stimuli for a brief duration followed by a mask such that the stimulus is consciously perceived on approximately 50% of trials (Koivisto et al., 2008). This approach allows comparisons of brain activity on aware vs. unaware trials while the physical stimuli (stimulus + mask) remain identical. Of course, with this approach all stimuli must be task-relevant because the experimenter has no other means of sorting individual trials into aware and unaware conditions. A different approach is to employ two different masking latencies (i.e., stimulus durations), one that is very short leading to 0% awareness and another that is considerably longer resulting in 100% awareness (Koivisto and Revonsuo, 2008). In this variation of the masking paradigm, task-relevance can be manipulated, and trial-by-trial reporting is unnecessary. However, the use of two different mask-onset latencies introduces another problem, namely that brain activity is likely to differ due to physical stimulus differences as opposed to a difference in awareness (Bachmann, 2009). For example, ERPs elicited by a mask presented 16 ms after a stimulus will be superimposed with ERPs elicited by the stimulus, whereas a mask presented 300 ms after a stimulus will only affect the stimulus-elicited ERPs at latencies beyond ~350 ms. To control for this confound, a control stimulus can be presented and masked at each of the two latencies, and the ERPs elicited by the control stimuli can be subtracted from the ERPs elicited by the stimuli of interest prior to making any aware vs. unaware contrasts. The subtraction essentially removes the mask-elicited ERP that is superimposed with the stimulus-elicited ERP. This was the approach used in the current study, which compared stimuli of 16 ms (unaware) vs. 300 ms (aware) duration, each of which was either task-relevant or task-irrelevant on separate blocks of trials. The overall design was similar to a previous study that compared ERPs elicited by masked vs. unmasked letters while spatial and non-spatial attention were manipulated (Koivisto and Revonsuo, 2007).

**Figure 3:**
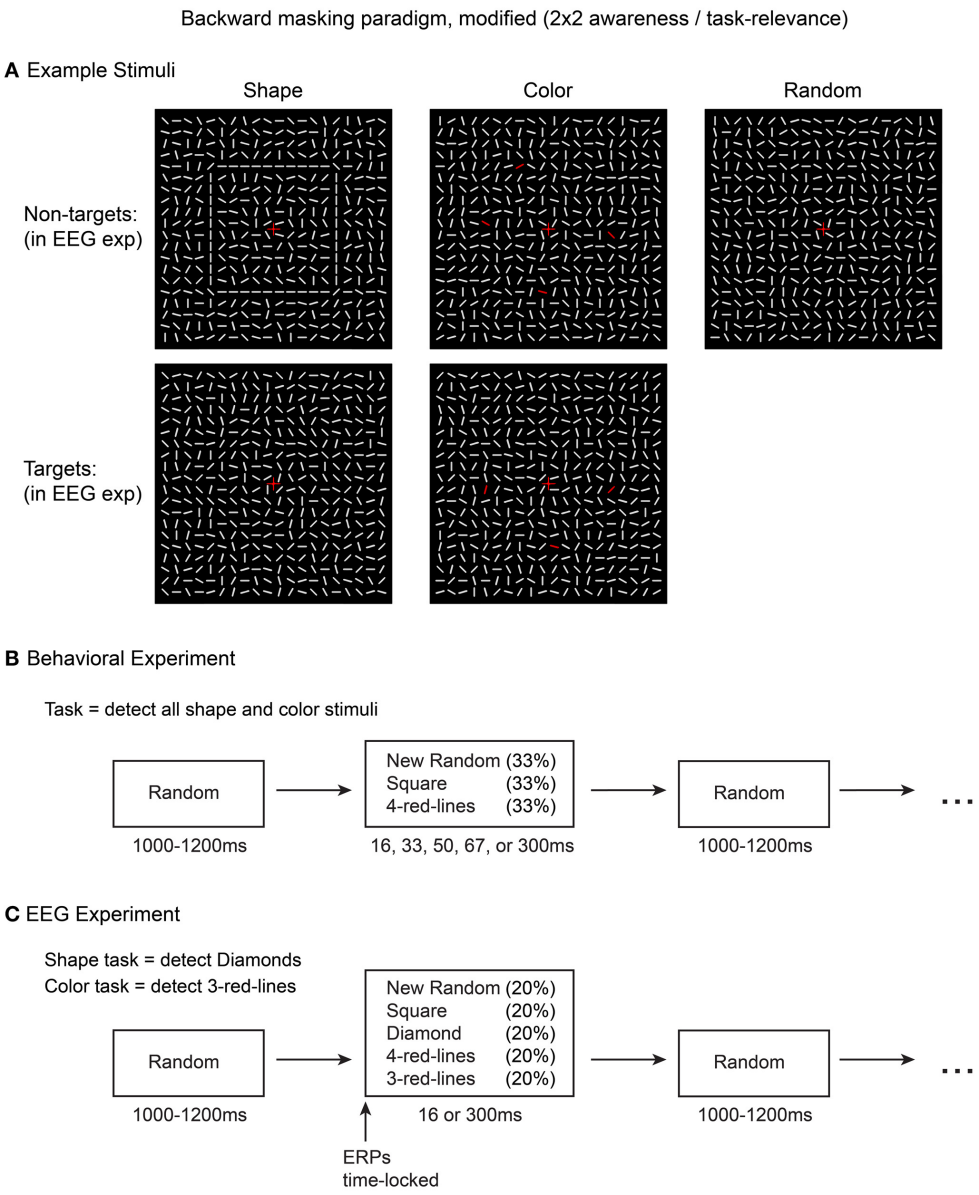
**Stimuli and methods used in the current experiment**. Five types of stimuli **(A)** were presented in the EEG experiment **(C)** while awareness was manipulated via short vs. long masking SOAs (16 or 300 ms). Task-relevance of the square and 4-red-line stimuli was manipulated by altering the target detection task. An initial behavioral experiment **(B)** was conducted to evaluate stimulus visibility at five different masking SOAs.

Here, the stimuli were contour shapes, colored lines, and control stimuli consisting of a random array of lines (Figure 3A). We first performed a behavioral detection study in which these stimuli were masked at 5 different latencies (i.e., stimulus durations of 16, 33, 50, 67, and 300 ms). Based on the results from this experiment, two masking latencies (16 and 300 ms) were selected for the EEG experiment because these mask-onsets showed close to 0 and 100% detection rates, respectively, for both shape and color stimuli. In the EEG experiment, on separate blocks of trials, shapes or colored-lines were deemed task-relevant, and the shape, color, and control stimuli were masked at each of the two latencies. This design allowed comparisons of ERP difference waves (shape minus control, color minus control) across 4 types of trials: aware, task-relevant; aware, task-irrelevant; unaware, task-relevant; unaware, task-irrelevant.

## Methods

### Participants

Twenty-six healthy adults participated in the EEG experiment. All were recruited as volunteers and gave informed consent prior to the beginning of the experiment. Data from eight participants were later excluded; three due to excessive EEG artifact, and five due to awareness of some of the 16 ms color stimuli, as assessed by a behavioral post-test (see below). The final group consisted of 18 participants (mean age: 21 years old; 13 female). An initial behavioral experiment was conducted to determine the masking SOAs used in the EEG experiment. Twelve (different) subjects participated in this behavioral study; data from one subject was excluded from analyses due to failure to follow task instructions. All experimental procedures were approved by the Reed College institutional review board in compliance with the Declaration of Helsinki.

### Stimuli and procedure

Stimuli consisted of 20 × 20 arrays (visual angle = 6.2 × 6.2°) of oriented white line segments (0.34°), identical to those used in Pitts et al. (2012, 2014). In the inter-stimulus intervals (ISIs) the lines were visible and in randomized orientations. Upon each stimulus presentation all of the lines rotated to new positions for the specified duration. For control stimuli, the line elements shifted to new random orientations. Two types of shape stimuli were created by arranging the new orientations of subsets of line segments to form a 10 × 10 square or a 7 × 7 diamond. Two types of color stimuli were created by changing either 3 or 4 of the randomly oriented line segments from white to red (RGB: 255,0,0). The spatial locations of the red lines were randomized but constrained to overlap with the locations of the shape contours to control for spatial attention across the shape and color tasks (see procedure below). Across trials, the positions of the red lines varied but were restricted to occur in upper, lower, left, and right regions with no two red lines being located in the same region. Example stimuli are shown in Figure 3A. For both the behavioral experiment and the EEG experiment, the line element arrays were always visible; no blank inter-stimulus intervals (ISIs) were used. Each stimulus presentation was followed by a return to a new pattern of randomly oriented line segments, which served as the masking/ISI stimuli; each ISI lasted a random interval between 1000 and 1200 ms. A central fixation cross (0.5°) was present at all times. All stimuli were presented on a dark background (0.07 cd/m2) on an LCD monitor (refresh rate = 60 Hz). Stimuli were created and displayed using Presentation software (Neurobehavioral Systems, Albany, CA).

In the behavioral experiment (Figure 3B), three different stimuli were presented: square shapes, 4-red-lines, and random (control) arrays. The diamond and 3-red-line stimuli were not presented because these stimuli would eventually serve as targets in the EEG experiment, while the ERPs of interest would be for the non-target stimuli (squares, 4-red-lines). Each stimulus was followed by a masking (ISI) stimulus at 5 equiprobable stimulus onset asynchronies (SOA): 16.67, 33.34, 50, 66.67, and 300 ms. Thus, there were 15 combinations of stimulus-type and mask SOA, and each combination was presented 20 times. The subject's task was to press a response button with their right index finger whenever they perceived either color or shape. Subjects were encouraged to adopt a liberal response criterion, responding even if they just caught a glimpse of color or shape, while avoiding guessing. The goal of this experiment was to determine a masking SOA that would render both color and shape invisible for all subjects on all trials. It was also important to verify that the 300 ms stimuli could be detected on 100% of trials.

Based on the results from the behavioral experiment, two different masking SOAs were employed in the EEG experiment (16.67 and 300 ms), resulting in 10 different stimulus-mask combinations: (2 types of shape stimuli + 2 types of color stimuli + 1 random control stimulus) × (2 mask SOAs) = 10 combinations. During each block of trials, these 10 trial types were intermixed and presented in random order, each at 10% probability. On separate blocks of trials, subjects performed either a color or a shape task. In the color task, the target stimuli were 3-red-lines and in the shape task, the diamond stimuli served as targets. Subjects pressed a response button with their right index finger upon target detection. In order to control for between-condition ERP differences associated with motor preparation and execution, all target trials (and any trials in which subjects responded) were excluded from ERP analyses. Each task was performed for 900 trials (~15 min) before switching to the other task for 900 trials, and this sequence was then repeated (task-order was counterbalanced across subjects). For example, half of the subjects performed the color task for 15 min, switched to the shape task for 15 min, switched back to the color task for 15 min, and finished with 15 min of the shape task. Thus, 1800 total trials were completed for each task, 180 of each stimulus type. Figure 3C shows a summary of the EEG experiment design, and a video example of the stimulus sequence is provided in Movie 1.

Because the behavioral experiment revealed detection rates greater than zero for the 16.67 ms color stimuli in some subjects, an additional behavioral post-test was conducted after each EEG session. In this post-test, color stimuli (4-red-lines), shape stimuli (squares), and control stimuli (random arrays) were presented and masked at 16.67 or 300 ms. A total of 180 stimuli were presented (across three 1 min blocks), 30 trials of each stimulus-mask combination. As in the behavioral study, subjects were instructed to press a button whenever they detected either color or shape (employing a liberal response bias). Five of the 26 participants detected 16.67 ms color stimuli on at least one trial during this post-test and were excluded from ERP analyses. No subjects detected any of the 16.67 ms shape stimuli, nor were there any “false alarm” responses to the randomized control stimuli. Active experiment time for the EEG study was ~60 min, short rest breaks were provided after every 60 trials (~1 min), and longer breaks were given after every 300 trials (~5 min). Each experimental session lasted 3.5–4.5 h including EEG cap preparation, practice trials, rest breaks, and the behavioral post-test.

### EEG recording and ERP pre-processing

EEG was noninvasively recorded from the scalp via Ag/AgCl electrodes sewn into customized caps with 96 electrode placements (EASYCAP, Herrsching, Germany). Electrode locations were modified from the standard 10–20 system to allow equidistant spacing (electrode positions reported here refer to the nearest channels of the international 10–20 system). Electrode impedances were kept below 5 kΩ. Signals were digitized (at 500 Hz) and amplified by three 32 channel amplifiers (Brain Amp Standard, Brain Products, Gilching, Germany). Eye movements and blinks were monitored by left and right horizontal EOG channels and a vertical EOG channel under the left eye, respectively. An electrode positioned at CPz served as the reference during recording.

ERPs were time-locked to the line segment orientation changes, low-pass filtered at 25 Hz (24dB/Oct), re-referenced to the average of the left and right mastoids, and baseline corrected from −100 to 0 ms. The left and right horizontal EOG channels were re-referenced as a bipolar pair. Trials were discarded if they contained eye movements, blinks, or other muscle artifacts in a −600 to +600 ms interval surrounding stimulus-onset. Artifact detection was accomplished semi-automatically via per-subject adjustment of the following peak-to-peak thresholds: eye movements (50 μV, 50 ms steps, in bipolar HEOG), blinks (100 μV, 200 ms steps, in VEOG and FP1), and muscle noise (150 μV, 200 ms steps, all remaining channels). On average, 16% of trials were rejected due to a combination of these artifacts. Individual electrodes showing extended periods of noise in the raw EEG were removed and replaced by interpolated signals from surrounding channels using topographic spherical splines (channels included in ANOVAs were not interpolated).

### ERP analyses

Our strategy for ERP analyses was to first identify time windows and electrodes of interest in the grand-averaged difference waves of all conditions averaged together, using our previous results as a guide (Pitts et al., 2012). Peak latencies were identified and mean amplitudes were assessed in ± 20 ms time windows around these peaks (± 50 ms for the broad P3b). The electrode showing the maximal signal, along with 5–8 adjacent sites (according to each component's scalp topography), were selected for analysis. For the shape stimulus difference waves (shape minus random), four distinct components were evident, each of which closely matched our previous results in terms of timing and scalp distribution: contour integration negativity, “CIN” (160–200 ms), visual awareness negativity, “VAN” (200–240 ms), late occipital positivity, “LOP” (310–350 ms), and the “P3b” (380–480 ms). For the color stimulus difference waves (color minus random), four components were also evident, some of which showed reduced latencies compared to the corresponding shape-elicited components. The first color-elicited component showed a very similar time course and scalp topography to a previously reported color vs. non-color ERP difference (Schoenfeld et al., 2003). This component, which consisted of a midline ventral-posterior negativity accompanied by a vertex positivity from 130 to 170 ms, has previously been referred to as the “sensory effect of color” which we abbreviate here as “SEC” (Zinni et al., 2014). The SEC (130–170 ms) was followed by the VAN (200–240 ms), LOP (290–330 ms), and P3b (380–480 ms) components, each of which showed similar scalp topographies to their shape-elicited counterparts.

Statistical analyses began with 2 × 2 ANOVAs with the factors awareness (16 or 300 ms durations) and task-relevance (attended or unattended) for each difference wave component using the time windows specified above. Difference wave amplitudes were averaged across electrode clusters according to the scalp topographies of each component as follows: CIN [*PO4, P6, O2, PO8, O10, PO10*]; SEC [*FC1, FCZ, FC2, C1, CZ, C2, CPZ*]; VAN [*PO4, P6, O2, PO8, O10, PO10*]; LOP [*P1, PZ, P2, PO3, POZ, PO4, O1, OZ, O2*]; P3b [*CP1, CPZ, CP2, P1, PZ, P2, POZ*].

All main effects and interactions were then further explored by conducting cluster mass permutation tests on the difference amplitudes (shape minus random; color minus random) for each of the 4 conditions separately (*aware, task-relevant*; *aware, task-irrelevant*; *unaware, task-relevant*; *unaware, task-irrelevant*). To increase statistical power, separate tests were carried out for the early (100–300 ms) and late (300–600 ms) time windows and permutation analyses were restricted to 63 of the 96 electrodes (covering central, parietal, temporal, and occipital regions) based on a priori hypotheses regarding scalp topographies of each component. Thus, 6363 comparisons were made for the early time window and 9450 for the late time window. In all cases, two-tailed cluster mass permutation tests (Bullmore et al., 1999), with a family-wise alpha level of 0.05, were conducted using the original data and 2500 random within-subject permutations of the data. Electrodes within approximately 3.02 cm of one another were considered spatial neighbors. All cluster mass permutation analyses were carried out using the mass univariate ERP Toolbox (Groppe et al., 2011).

## Results

### Behavioral experiment

**Figure 4:**
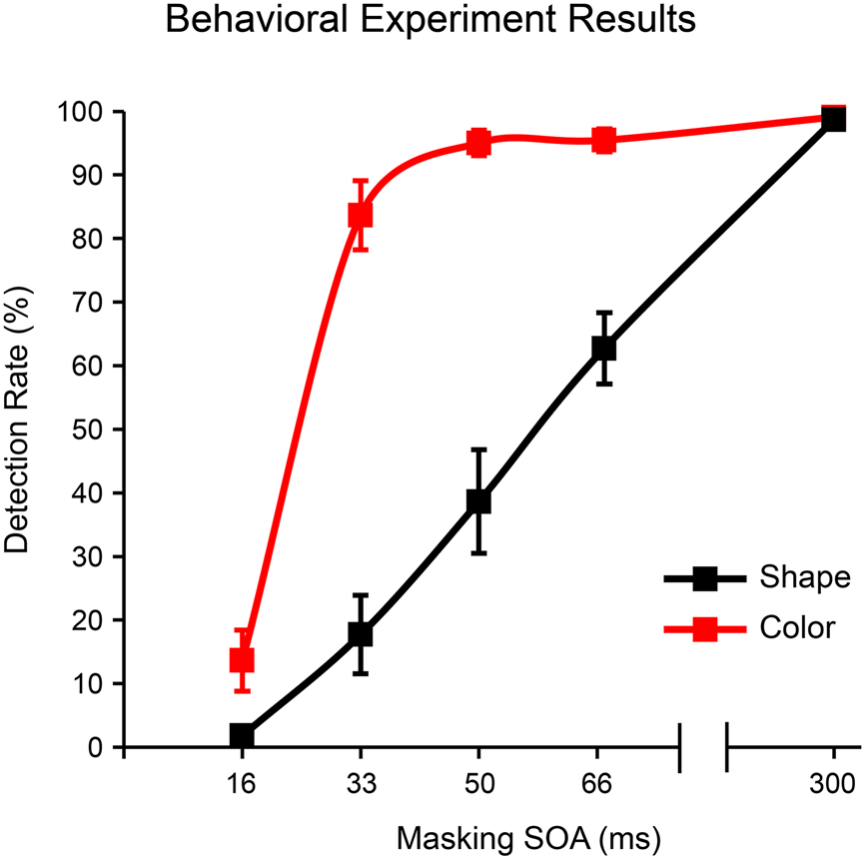
**Detection rates as a function of masking SOA for shape (square) and color (4-red-lines) stimuli in the behavioral experiment**. Error bars reflect the standard error of the mean (s.e.m.).

Mean shape and color detection rates at each of the five masking SOAs tested are provided in Figure 4. Overall, the shape stimulus was more readily masked than the color stimulus. Eight of the 11 subjects never responded to shapes presented for 16 ms, two subjects responded once (5%) and one subject twice (10%); note that some of these responses may have reflected accidental button presses rather than transient perceptual capacities. For the 16 ms color stimulus, five subjects never responded, and detection rates for the remaining six subjects were as follows: 10, 15, 25, 30, 30, 40%. The average false alarm rate (responses to random stimuli at any of the five stimulus durations) was 0.5%. Three subjects had 2% false alarm rates while all other subjects had zero false alarms. All three subjects showing false alarm rates > 0 also showed detection rates > 0 for the 16 ms color stimulus.

Because our goal was to identify a single masking SOA that could render both shape and color stimuli invisible for a large majority of subjects/trials, we chose the 16 ms SOA for use in the EEG experiment. Importantly, because roughly half of the subjects in this behavioral experiment showed non-zero detection rates for the 16 ms color stimulus, we also administered a behavioral post-test after each EEG session (see methods above). We excluded from EEG analyses any subject who responded to one or more 16 ms stimuli in this post-test (five out of 26 subjects were excluded for this reason). For the remaining subjects, detection rates in this behavioral post-test were 0% for both 16 ms stimuli and 97.73% (s.e.m. = 0.18%) and 98.19% (s.e.m. = 0.10%) for the 300 ms shape and color stimuli, respectively.

### EEG experiment

Behavioral results from the EEG experiment indicated that the shape task was slightly more difficult than the color task, although performance on both tasks was strong. For the shape task, subjects detected the 300 ms diamond-shaped targets on 93.55% of trials (s.e.m. = 1.3%), d' = 4.61 (s.e.m. = 0.11), RT = 563 ms (s.e.m. = 10 ms). For the color task, subjects detected the 300 ms 3-red-line targets on 98.24% of trials (s.e.m. = 0.5%), d' = 4.68 (s.e.m. = 0.09), RT = 537 ms (s.e.m. = 10 ms). While d' did not differ statistically across the two tasks, RTs were significantly shorter for the color task, *t*_(18)_ = 4.56, *p* = 0.0003. Response rates to the 16 ms color and 16 ms shape targets were both 0%.

**Figure 5:**
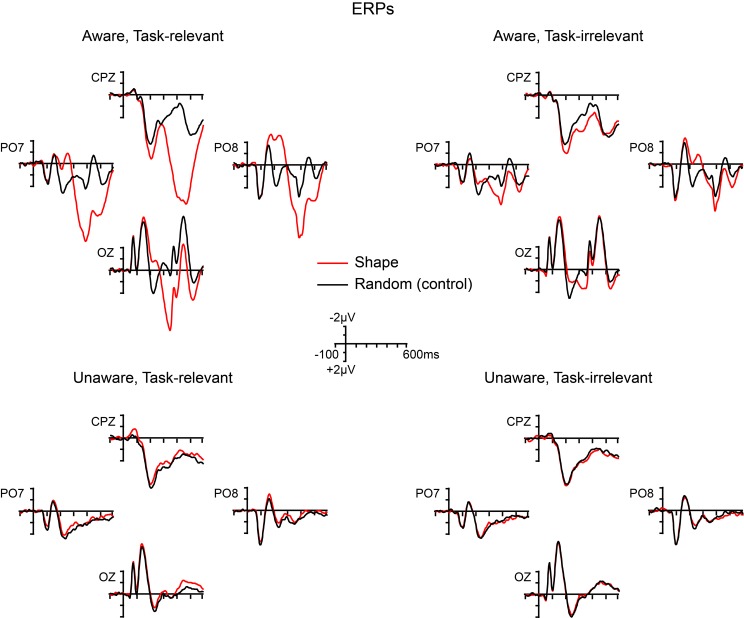
**Grand-averaged ERPs elicited by shape (square) and random (control) stimuli across the four main experimental conditions**.

**Figure 6:**
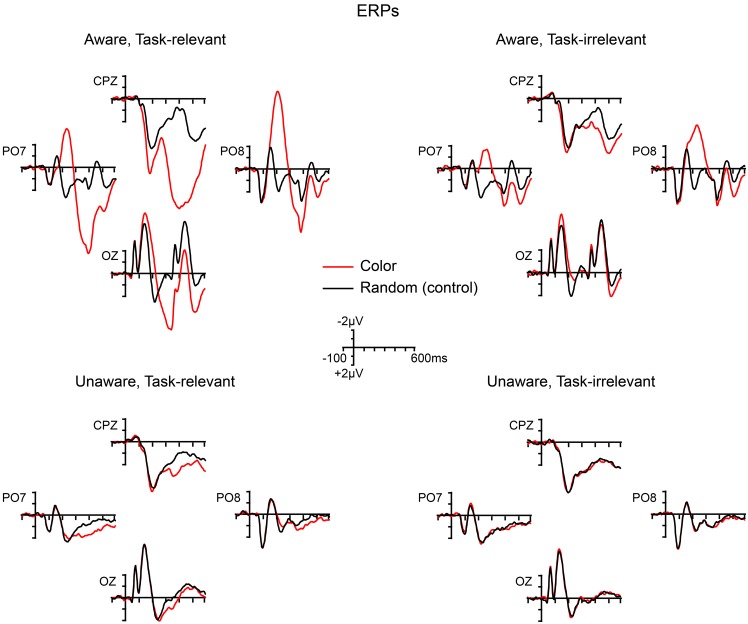
**Grand-averaged ERPs elicited by color (4-red-lines) and random (control) stimuli across the four main experimental conditions**.

**Figure 7:**
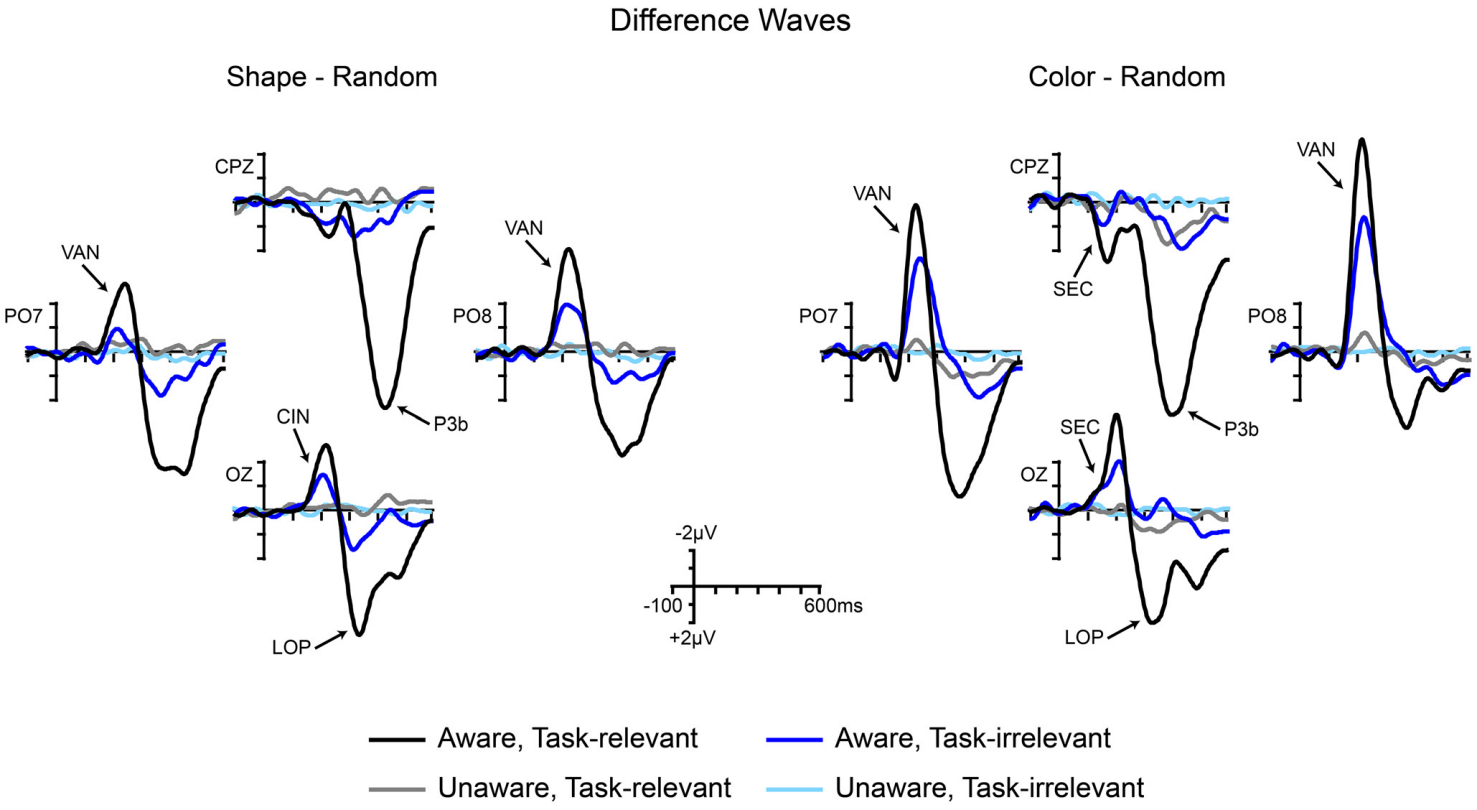
**Grand-averaged difference waves for shape stimuli (square minus random) and color stimuli (4-red-lines minus random) across the four conditions of interest**. Components are labeled at representative electrodes. CIN, contour-integration negativity; SEC, sensory effect of color; VAN, visual awareness negativity; LOP, late occipital positivity; P3b, centro-parietal positivity.

Grand-averaged ERPs elicited by the non-target shape and color stimuli are compared to ERPs elicited by the random (control) stimuli in Figures 5, 6, respectively. In both figures, electrodes representative of the scalp locations of the main components of interest are shown. Note that the ERPs elicited by the control stimuli differ according to stimulus duration and that within-block comparisons were made in all cases. For example, the shape-elicited ERPs in the aware, task-relevant condition were compared to the random-stimulus ERPs during the shape-task blocks, whereas the shape-elicited ERPs in the aware, task-irrelevant condition were compared to the random-stimulus ERPs during the color-task blocks. Difference waves formed by subtracting the ERPs to the appropriate control stimuli from the ERPs to the shape and color stimuli are shown in Figure 7. In these difference waves the components CIN (shape) SEC (color), VAN, LOP, and P3b can be visualized. Results from all statistical analyses are provided below for shape and color stimuli, for each of the components of interest, organized according to their temporal sequence (see Methods Section for descriptions of each component's time course and scalp distribution).

#### Early sensory effects

**Figure 8:**
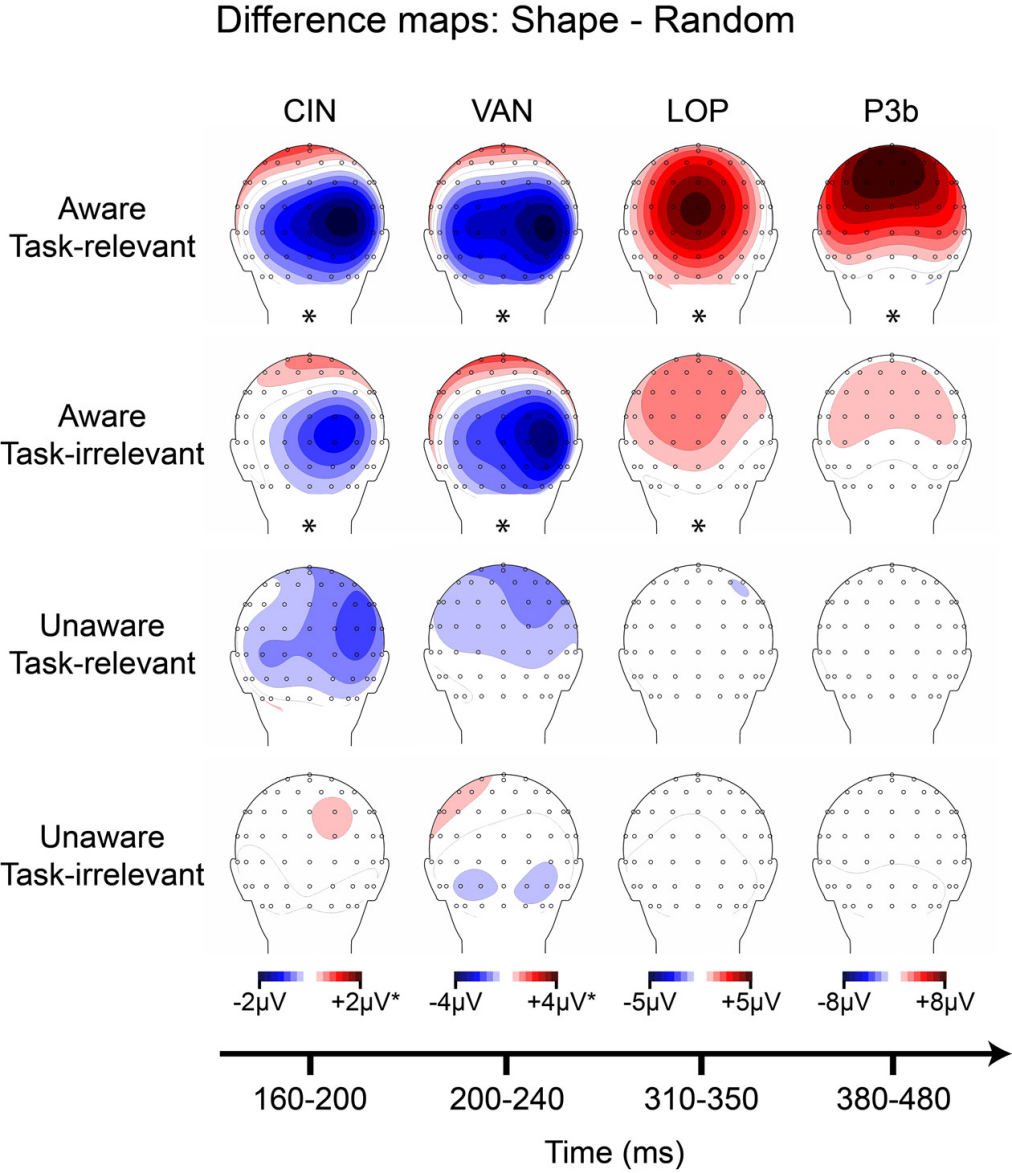
**Grand-averaged difference wave topographies (square minus random) plotted over the posterior scalp for the four components of interest (columns) across the four main experimental conditions (rows)**. Note that the amplitude scale for the CIN component was adjusted to ± 1 μV for the unaware conditions, and for the VAN component ± 2 μV for all conditions except the aware, task-relevant condition. Asterisks indicate significant amplitude differences (*p* < 0.05) between shape and random ERP as assessed via cluster mass permutation tests for each condition separately.

ANOVA for the CIN component elicited by the outline square showed a main effect of awareness, *F*_(17)_ = 12.42 (*p* = 0.0026), a main effect of task-relevance, *F*_(17)_ = 11.74 (*p* = 0.0032), and no interaction. Cluster mass permutation analyses for each condition separately confirmed that these main effects were due to significant amplitude differences in the *aware, task-relevant* (−1.58 μV, *SD* = 1.38) and *aware, task-irrelevant* (−0.91 μV, *SD* = 1.41) conditions (Figure S1). Difference amplitudes in the *unaware, task-relevant* and *unware, task-irrelevant* conditions did not significantly differ from zero; although a trend toward a negative difference over the posterior scalp was observed for the *unaware, task-relevant* conditions (see Figure 8).

ANOVA for the SEC component elicited by the colored lines revealed a main of effect of awareness, *F*_(17)_ = 15.24 (*p* = 0.0011), with no main effect of task-relevance, nor an interaction between the two. Permutation analyses confirmed significant amplitude differences in the *aware, task-relevant* (1.87 μV, *SD* = 2.16) and *aware, task-irrelevant* (0.83 μV, *SD* = 1.24) conditions, while amplitude differences in both unaware conditions were not significant (Figure S2).

#### Visual awareness negativity (VAN)

For the shape-elicited VAN component, ANOVA resulted in a main effect of awareness, *F*_(17)_ = 11.83 (*p* = 0.0031), a main effect of task-relevance, *F*_(17)_ = 17.83 (*p* = 0.00057), and a significant interaction, *F*_(17)_ = 8.42 (*p* = 0.0099). Cluster mass permutation tests revealed a significant VAN component in the *aware, task-relevant* and *aware, task-irrelevant* conditions and an absence of this component in both unaware conditions. The interaction between awareness and task-relevance was explained by a substantial amplitude increase in the *aware, task-relevant* (−3.19 μV, *SD* = 3.09) compared to the *aware, task-irrelevant* condition (−1.58 μV, *SD* = 2.02).

ANOVA for the color-elicited VAN showed a main effect of awareness, *F*_(17)_ = 40.91 (*p* = 0.000007), a main effect of task-relevance, *F*_(17)_ = 15.05 (*p* = 0.0012), and an interaction between the two *F*_(17)_ = 14.21 (*p* = 0.0015). Similar to the shape-elicited VAN, cluster permutation tests showed significant amplitude differences in both aware conditions and the interaction was explained by relatively larger amplitudes in the *aware, task-relevant* (−5.77 μV, *SD* = 3.91) vs. the *aware*, *task-irrelevant* condition (−3.56 μV, *SD* = 2.54) along with no significant differences in either of the unaware conditions.

#### Late positivites

ANOVA for the shape-elicited LOP component showed a main effect of awareness, *F*_(17)_ = 46.79 (*p* = 0.000003), a main effect of task-relevance, *F*_(17)_ = 11.03 (*p* = 0.0040), and a significant interaction, *F*_(17)_ = 29.60 (*p* = 0.000044). Permutation tests suggested that these effects were due to a large amplitude difference in the *aware, task-relevant* condition (4.48 μV, *SD* = 2.70), a smaller difference in the *aware, task-irrelevant* condition (1.48 μV, *SD* = 2.02), along with no significant amplitude differences in either unaware condition.

For the color-elicited LOP, ANOVA indicated a main effect of awareness, *F*_(17)_ = 15.67 (*p* = 0.0010), a main effect of task-relevance, *F*_(17)_ = 35.41 (*p* = 0.000016), and a significant interaction, *F*_(17)_ = 25.05 (*p* = 0.00011). However, unlike the shape-elicited LOP, cluster mass permutation tests showed that these effects were driven by a large amplitude difference in the *aware, task-relevant* condition (4.59 μV, *SD* = 3.48), along with a smaller effect in the *unaware, task-relevant* condition (0.67 μV, *SD* = 1.09). No amplitude differences during the LOP time window were evident in the *aware, task-irrelevant* or *unaware, task-irrelevant* conditions.

Finally, for the P3b analyses, ANOVA for the shape stimuli resulted in a main effect of awareness, *F*_(17)_ = 26.67 (*p* = 0.000078), a main effect of task-relevance, *F*_(17)_ = 18.51 (*p* = 0.00048), and a significant interaction, *F*_(17)_ = 36.85 (*p* = 0.000012). All of these effects were driven by a large amplitude difference in the *aware, task-relevant* condition (7.72 μV, *SD* = 5.85), along with no significant amplitude effects in any of the other conditions. Although cluster permutation analyses showed positive amplitude differences in the *aware, task-irrelevant* condition from ~300–400 ms (Figure S1), these effects correspond to the small LOP described above and were absent for electrode sites and time windows corresponding to the P3b.

ANOVA for the P3b elicited by the color stimuli also showed a main effect of awareness, *F*_(17)_ = 33.32 (*p* = 0.000023), a main effect of task-relevance, *F*_(17)_ = 34.71 (*p* = 0.000018), and a significant interaction, *F*_(17)_ = 22.63 (*p* = 0.00018). Cluster mass permutation tests for each condition revealed a different pattern of effects from that of the shape-elicited P3b. In this case, color-elicited P3b amplitudes were significant for all conditions except the *unaware, task-irrelevant* condition. The P3b in the *unaware, task-relevant* condition (1.31 μV, *SD* = 1.43) was similar in magnitude to the P3b in the *aware, task-irrelevant* condition (1.73 μV, *SD* = 1.76) and occurred slightly earlier in time (see Figure S2), while the P3b in the *aware, task-relevant* condition was clearly the largest (8.28 μV, *SD* = 5.50).

#### Results summary

**Figure 9:**
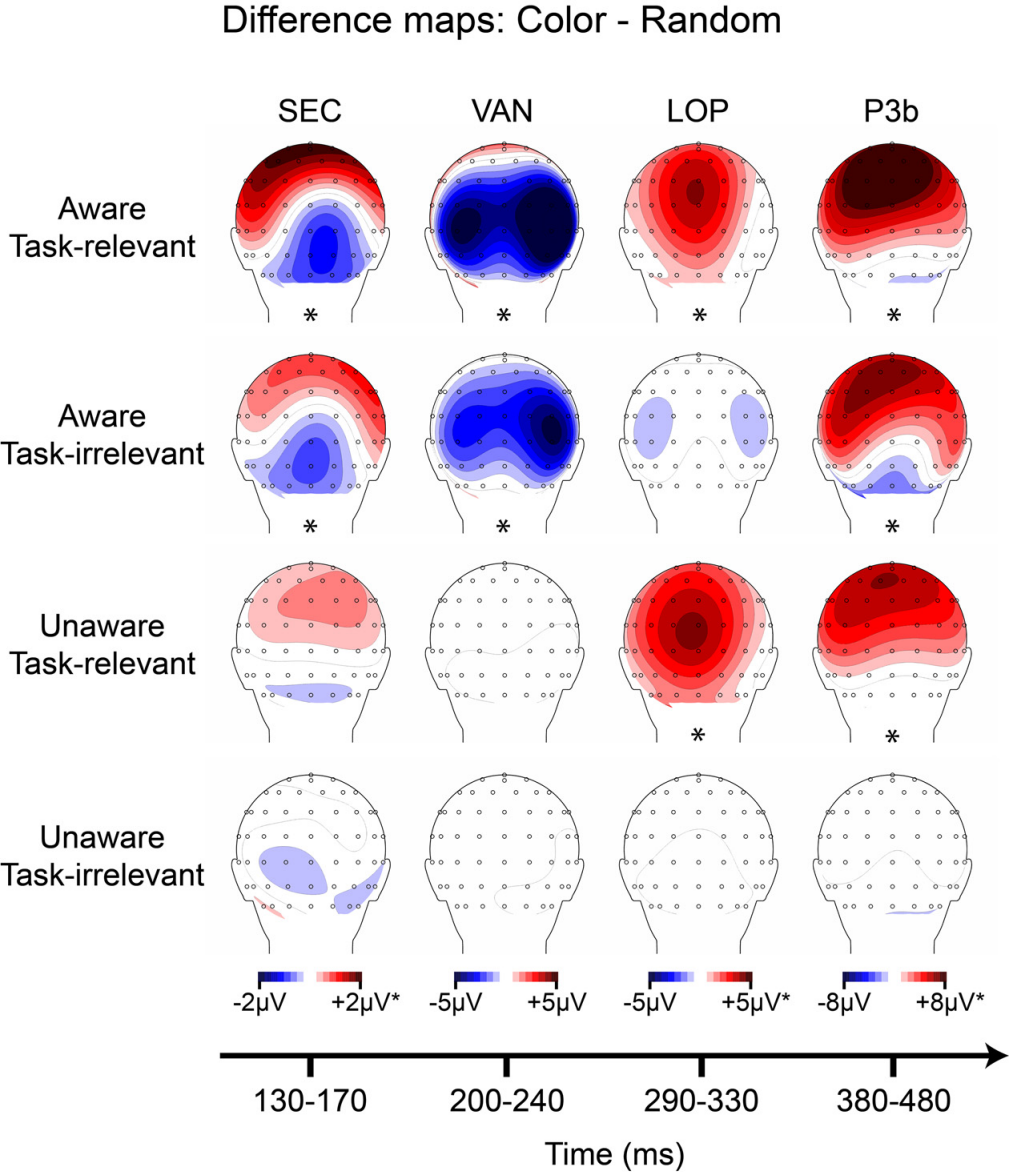
**Grand-averaged difference wave topographies (4-red-lines minus random) plotted over the posterior scalp for the four components of interest (columns) across the four main experimental conditions (rows)**. Note that the amplitude scales for the SEC and LOP components were adjusted to ± 1 μV for the unaware conditions, and for the P3b component ± 2 μV for all conditions except the aware, task-relevant condition. Asterisks indicate significant amplitude differences (*p* < 0.05) between shape and random ERP as assessed via cluster mass permutation tests for each condition separately.

Figures 8, 9 provide summaries of the results for shape and color stimuli, respectively. In these figures, scalp topographies for each of the main components (defined by amplitude differences between shape and random ERPs, and color and random ERPs, respectively) are provided across all four conditions resulting from the 2 × 2 manipulation of awareness and task-relevance. Asterisks under the scalp maps indicate that a significant amplitude difference was found during the component's time-window as assessed by cluster mass permutation tests.

The overall goal of this experiment was to identify components that are present in both aware conditions (regardless of task-relevance) and absent in both unaware conditions. Across the shape and color stimuli, only the early sensory components (CIN, SEC) and the visual awareness negativity (VAN) showed this pattern. For both shape and color, these components were evident in aware conditions and absent in unaware conditions, regardless of task-relevance. Whereas the LOP also showed this pattern for the shape stimuli (Figure 8), it was absent for aware, task-irrelevant color stimuli (Figure 9). The P3b did not consistently correlate with awareness in shape or color trials (Figures 8, 9), being absent for aware, task-irrelevant shapes, and present for unaware, task-relevant color.

## Discussion

In agreement with previous results using the inattentional blindness paradigm (Pitts et al., 2012, 2014), the present study used a 2 × 2 manipulation of awareness and task-relevance in a backward masking task and found a consistent ERP correlate of conscious visual perception, the visual awareness negativity (VAN) component. The VAN is a mid-latency occipital-parietal negativity (measured here from 200 to 240 ms) that has been targeted by a number of previous studies using various awareness manipulations (Koivisto and Revonsuo, 2003, 2008, 2010; Ojanen et al., 2003; Wilenius-Emet et al., 2004; Koivisto et al., 2005; Wilenius and Revonsuo, 2007; Railo et al., 2011; Sandberg et al., 2013). ERP components preceding the VAN are likely to reflect pre-conscious processing, considering that a previous study using identical stimuli (Pitts et al., 2012) found evidence for the elicitation of such components in unaware (and unmasked) conditions. In the current study, components prior to the VAN were absent in the unaware conditions, suggesting interference of pre-conscious processing by the masking stimulus (presented at 16 ms SOA). Components subsequent to the VAN have often been proposed as potential NCCs, including most notably the P3b component (Sergent et al., 2005; Babiloni et al., 2006; Del Cul et al., 2007; Lamy et al., 2009; Dehaene and Changeux, 2011; Batterink et al., 2012; Kouider et al., 2013). The current study, however, provides evidence against this view, as the P3b along with another component subsequent to the VAN (the LOP) did not consistently correlate with awareness. Instead, these later components are likely to reflect post-perceptual or attention-based processes necessary for completing the task, as their amplitudes were by far the largest in the aware/task-relevant condition. Moreover the P3b was present in some of the unaware, task-relevant conditions, and absent in some of the aware, task-irrelevant conditions.

### Correlates of visual awareness vs. post-perceptual processing

It is difficult to create controlled conditions in which subjects are aware vs. unaware of physically identical stimuli without requiring an immediate trial-by-trial perceptual report. Nevertheless, it is important to devise new methods for allowing such contrasts because the neural correlates of conscious perception are easily confusable with the neural correlates of post-perceptual maintenance and access for immediate subjective report. In both task-relevant and task-irrelevant situations (in which the stimuli are readily visible), subjects are considered to be conscious of the perceptual information because whenever they are asked to report what they see, they are able to easily do so. It follows that brain activity associated with conscious perception should be present in both cases. However, when perceptual information is accessed for immediate report, a variety of additional neural processes are likely to ensue, each of which is related to “doing something extra” with the perceptual information the subject is already conscious of. Importantly, our manipulation of task-relevance was designed to test a particular post-perceptual process: *access of perceptual information for report*. This should not be confused with the more general concept of *conscious access* (Dehaene and Changeux, 2004; Block, 2005), which we assume was present in our aware (300 ms stimulus) task-*irrelevant* conditions. In other words, we expect that on any given trial, if the subjects had been asked, they would have easily been able to report seeing the 300 ms task-irrelevant stimuli (this was indirectly verified by our behavioral experiment and behavioral post-test), thus perceptual information was globally available but the subjects did not have to use this information to complete the primary task-at-hand.

In our previous inattentional blindness experiments (Pitts et al., 2012, 2014; Shafto and Pitts, 2013), we attempted to deal with this issue by rendering a stimulus easily visible (if expected) but irrelevant to the task-at-hand and delaying the perceptual report until after a full block of trials. In this situation, in which subjects were fully aware of a stimulus but did not have to do anything with the perceptual information because it was irrelevant to the task, we found that only early sensory (CIN) and mid-latency (VAN) ERP components were elicited. Subsequent components such as the P3b were only evident when the stimuli became relevant to the task. In the current experiment, instead of diverting attention to create an unaware condition, we severely masked some stimuli (16 ms masking SOA) and compared ERPs to conditions in which the same stimuli were clearly visible (300 ms masking SOA). Importantly, in order to assess post-perceptual processing we manipulated the task such that stimuli were relevant to the task during some blocks of trials and irrelevant during other blocks. We found a similar pattern of results as in the inattentional blindness studies, in that the VAN was consistently associated with perceptual awareness, while subsequent components were either absent when the stimuli were irrelevant to the task or present when subjects were unaware of task-relevant stimuli.

Despite the main pattern of results, it remains possible that a small P3b was present (but below the statistical threshold) during aware, task-irrelevant shape trials (both in the current study as well as in Pitts et al., 2012 and Pitts et al., 2014). In this case, one might argue that the P3b is a correlate of conscious perception but was very weak during the task-irrelevant conditions because subjects were so focused on the relevant stimuli that they only caught a glimpse of the irrelevant stimuli on a subset of trials. Future studies might address this by incorporating infrequent, unexpected, probe trials in which the experiment is interrupted after a task-irrelevant stimulus and subjects are asked to “report what you just saw.” If subjects can always report awareness of task-irrelevant stimuli, but the P3b is still absent, a stronger case could be made for a lack of a relationship between the P3b and awareness; however, one must be careful to avoid inadvertently rendering the task-irrelevant stimuli relevant by including too many “surprise” report trials.

### Attention in the absence of awareness?

An unexpected finding in the current study was evidence for a P3b elicited in the absence of reported awareness. This effect appeared in the unaware, task-relevant condition for color stimuli. It was absent for the equivalent condition for shape stimuli and there was no evidence for a P3b in any of the unaware, task-irrelevant conditions. While very few studies have reported a P3b in the absence of conscious perception, it is generally accepted that attention and awareness are independent (yet often interact), and there is growing evidence for attentional modulations in the absence of awareness (Bernat et al., 2001; Koch and Tsuchiya, 2007; Wyart and Tallon-Baudry, 2008; Tsuchiya and Koch, 2009; Bachmann, 2011; Tallon-Baudry, 2011; Marchetti, 2012; Aru and Bachmann, 2013). We previously posited that the P3b is post-perceptual and argued that while the P3b does not appear to be necessary for conscious perception, it may be a sufficient marker; i.e., whenever a P3b is observed one can be confident that the subject was aware of the stimulus (Pitts et al., 2012, 2014). The current result, however, suggests that the P3b might not be necessary *or* sufficient, as it was elicited by color stimuli of which the subject was presumably unaware. Interestingly, the P3b was completely absent for task-irrelevant color stimuli of which the subject was also unaware, thus suggesting a task-based attentional modulation of perceptually undetected stimuli. This result is consistent with a recent study that showed an N2pc component for color singletons that captured attention even when the subject was unaware of the stimuli, but only when color was relevant to the search task (Ansorge et al., 2010), as well as an earlier study that found evidence of attention capture (N2pc) in the absence of awareness due to object substitution masking (Woodman and Luck, 2003). Similarly, recent studies have provided evidence for working memory related processing in the absence of awareness (Hassin et al., 2009; Gilchrist and Cowan, 2010; Soto et al., 2011).

An alternative explanation for this finding is that some subjects on some trials may have been partially aware of the 16 ms color stimuli even though their reports indicated lack of awareness. In these partial awareness situations, subjects may have attempted to perform the discrimination task (to determine if the stimulus contained 3 or 4 red lines) but could only detect the presence of color without being able to discriminate between target and non-target color stimuli (see Windey et al., 2014 for a recent review of graded vs. dichotomous awareness). While we conducted an initial behavioral study to determine an appropriate masking SOA and administered a behavioral post-test to each of the EEG subjects in which a simple detection task replaced the discrimination task, it is still possible that some residual awareness for a number of color stimuli occurred during the EEG portion of the study. Indeed, we excluded five out of the original 26 participants because they were able to detect at least one of the 16 ms color stimuli during the behavioral post-test. Because one of the goals of the current design was to avoid trial-by-trial reports, we intentionally did not acquire the data necessary to fully evaluate this alternative interpretation. Future studies should consider following up on this preliminary result in order to determine whether a P3b can be elicited during attentive, but unaware conditions, perhaps by employing a detection task with low response criterion during EEG recording (Squires et al., 1973, 1975).

### Pre-conscious processing

In contrast to our previous results using the inattentional blindness paradigm (Pitts et al., 2012), the current study provided no evidence for an early pre-conscious ERP difference between shape and random stimuli in the unaware conditions. One of the major findings in our previous study was that a component we labeled as Nd1 (for negative difference 1, here referred to as the CIN), distributed over the occipital midline from ~160–200 ms, was elicited in all conditions, even when subjects were unaware of (inattentionally blind to) the shape patterns. Why was this component absent from the unaware conditions in the current study? The most likely possibility is due to the differences in bottom-up stimulus strength between the two studies, a factor that is known to influence whether a stimulus will be processed non-consciously, pre-consciously, or consciously (Dehaene et al., 2006). In our previous inattentional blindness experiment, the shape stimuli were always presented for 300 ms in duration (including the unaware condition), whereas the current study employed heavily masked stimuli (16 ms durations) to create the unaware conditions. In line with the interactions between top-down attention and bottom-up stimulus strength described by Dehaene et al. (2006), the current results showed a small (just below statistical threshold) CIN in the unaware, task-relevant condition. In other words, with such severe masking and the resulting reduction of bottom-up stimulus strength, an unseen stimulus might only be processed pre-consciously if attended. In contrast, in our previous study top-down attention was not required because sufficient bottom-up stimulus strength allowed this same stimulus to be processed pre-consciously.

One of the advantages of the current experimental design was a degree of internal replication made possible by the 2 × 2 crossing of the color and shape stimuli/tasks. This allows one to ask whether the same pattern of results seen for the shape stimuli was also observed for the color stimuli. An early sensory effect of color (SEC) was not observed for either of the unaware conditions, although a trend in this direction was evident (see Figure 9, left column, third row). To further explore how pre-conscious processing is influenced by top-down attention and bottom-up stimulus strength, future studies could present extended duration color stimuli during inattentional blindness or present backward-masked color stimuli at various masking SOAs while manipulating task-relevance.

### VAN: attention or awareness?

A main focus of recent consciousness research has been the relationship between attention and awareness (Tsuchiya and van Boxtel, 2013). Historically, attention and consciousness were often treated as similar if not identical concepts; nowadays, however, many researchers are proposing that each refers to a separate category of neural and psychological processes. Although attention and awareness may be functionally distinct, the question of whether each can exist independently of the other remains a topic of debate (Cohen et al., 2011, 2012; Tsuchiya et al., 2012; Aru and Bachmann, 2013). One view posits that attention can operate in the absence of awareness, and awareness can occur in the absence of attention, whereas an opposing view argues that while attention can influence processing of stimuli of which the subject is unaware, there is no such thing as awareness in the absence of attention; i.e., attention is necessary for conscious perception. Results from experiments employing the inattentional blindness paradigm offer strong support for the latter view (Cohen et al., 2011; Mack and Clarke, 2012; Pitts et al., 2012).

The present results, along with our previous inattentional blindness results, suggest that the most viable candidate for an ERP correlate of awareness is the VAN (Nd2) component. In our previous study (Pitts et al., 2012), a well-known attention-related component, the SN (Harter and Aine, 1984; Hillyard and Anllo-Vento, 1998), was evident immediately after the VAN, but was only observed in conditions where the stimulus was task-relevant. Distinguishing between the VAN and the SN in a data-driven manner is not easy, because both components consist of a mid-latency (~200–300 ms), posterior, bi-lateral negativity. To further complicate matters, both the VAN and the SN show variable (and overlapping) latencies depending on the stimuli and task. The only way to isolate the VAN from the SN component may be to manipulate task-relevance; in our previous study both components were present in task-relevant situations, whereas only the VAN was present in aware but task-irrelevant conditions (Pitts et al., 2012). In the present study, however, no clear SN was evident in the aware, task-relevant condition, perhaps because the stimuli used here were so easily discriminable that the processing resources indexed by the SN did not have to be engaged. Alternatively, it is possible that the VAN measured in the current study overlapped the SN in the task-relevant condition such that the two components were indistinguishable. It will be important for future studies to distinguish between the VAN and the SN, especially in situations in which the stimuli are task-relevant.

In the current study as well as in Pitts et al. (2012), the amplitude of the VAN was larger in the task-relevant compared to the task-irrelevant condition. If the VAN indeed reflects perceptual awareness, which is often assumed to be an all-or-none phenomenon (you see the stimulus or you don't), why might its amplitude vary according to the task? One possibility might be that subjects were not aware of the task-irrelevant 300 ms stimuli on every single trial because their attention was focused on a separate task (i.e., partial inattentional blindness). This seems unlikely, especially for the color stimuli (see Movie 1); in a separate experiment (Pitts et al., 2014) we found a 0% inattentional blindness rate for similar 300 ms color stimuli. Another possibility is that the timing of perceptual awareness is more consistent across trials when the stimulus is relevant to the task; whereas, on task-irrelevant trials subjects may notice the irrelevant stimuli on every trial but at slightly different times on different trials because their primary task is to determine if a potential target is present. If this is the case, one would expect larger amplitudes with briefer time-courses for awareness-related ERPs in task-relevant conditions and smaller amplitudes with extended durations for the same ERP components in task-irrelevant conditions, given that the ERPs are derived by averaging across many trials. Data from Pitts et al. (2012) follow this pattern very closely, and although the current data show amplitude differences for the VAN without obvious corresponding differences in component duration, the shape of the VAN is slightly skewed with a longer right-tail in the task-irrelevant condition (see Figure 7), consistent with the trial-by-trial latency jitter account. A third alternative explanation is that the VAN and SN components may have been temporally superimposed such that only a portion of this negativity reflects perceptual awareness. The amplitude increase during task-relevant situations might be due to an increase in top-down attention reflected by the SN component, rather than a larger number of aware trials or greater trial-to-trial consistency in the timing of awareness.

While there is growing evidence that the VAN tracks closely with awareness while the SN varies as a function of task demands and attention, it is important to acknowledge the existence of a variety of attentional processes. In addition to exogenous (bottom-up) and endogenous (top-down) attention, visual attention can be selectively allocated to spatial locations, features, or entire objects. An alternative interpretation of the VAN is that it reflects some form of object-based attention that is necessary for conscious perception (perhaps a specific interaction between attention and high-level perceptual representations). Because many manipulations of awareness also involve manipulations of attention, this possibility cannot be easily discounted. To give some examples, inattentional blindness involves altering attention to alter awareness; backward masking at short vs. long latencies alters bottom-up attention to influence awareness; backward masking and signal detection at threshold capitalizes on stochastic trial-by-trial fluctuations of attention; the attentional blink involves differences in attention between seen and unseen stimuli; and change blindness differs from change detection based on the allocation of spatial attention. Because of the common co-manipulation of attention and awareness across a variety of paradigms, we previously argued that naming an ERP component the “visual awareness negativity” (VAN) might be pre-mature (Pitts et al., 2012), although we adopt this nomenclature here for consistency with the literature. If the VAN turns out to reflect a type of object-based attention instead of awareness *per se*, this might suggest that an obvious ERP correlate of conscious perception has yet to be discovered (Verleger, 2010). This would explain the amplitude increase for the VAN in task-relevant vs. task-irrelevant conditions, i.e., task-relevance enhances object-based attention. Importantly, this would not mean that a neural correlate of awareness does not exist, but rather that ERPs can only measure a limited set of neuronal events. In any case, studies of ERPs can still be useful in helping to narrow down the time-window for which potential NCCs could be found, while more sensitive techniques such as intra-cranial recordings in human epileptic patients may be necessary to identify NCCs.

A final possibility worth consideration is whether a particular type of interaction between attention and perceptual representation *is* the underlying neural mechanism of conscious awareness, and the VAN is a marker of this type of interaction. To explore this idea, future consciousness research might focus efforts on understanding object-based attention, perceptual encoding, and the interaction between the two, rather than searching for neurons or neural networks specifically dedicated to consciousness *per se* (Cohen and Dennett, 2011). Currently, there is not strong evidence for or against the view that the VAN component reflects object-based attention instead of visual awareness. In addition to developing experimental paradigms which can better isolate NCC-proper from pre-conscious and post-perceptual activity (the focus of the current special issue), it is imperative that researchers craft experimental designs that improve our chances of distinguishing between neural correlates of object-based attention and neural correlates of awareness.

### Conflict of interest statement

The authors declare that the research was conducted in the absence of any commercial or financial relationships that could be construed as a potential conflict of interest.

## Supplementary material

Movie 1 **Video depicting an example sequence of trials from the EEG experiment (instructions refer to a block of trials in which shapes were task-relevant)**.

## References

[B1] Ansorge U. Horstmann G. Worschech F. (2010). Attentional capture by masked colour singletons. Vision Res. 50, 2015–2027 10.1016/j.visres.2010.07.015 20659496

[B2] Aru J. Bachmann T. (2013). Phenomenal awareness can emerge without attention. Front. Hum. Neurosci. 7:891 10.3389/fnhum.2013.00891 24391577 PMC3868908

[B3] Aru J. Bachmann T. Singer W. Melloni L. (2012). Distilling the neural correlates of consciousness. Neurosci. Biobehav. Rev. 36, 737–746 10.1016/j.neubiorev.2011.12.003 22192881

[B4] Baars B. J. (1989). A Cognitive Theory of Consciousness. Cambridge, MA: Cambridge University Press

[B5] Babiloni C. Vecchio F. Miriello M. Romani G. L. Rossini P. M. (2006). Visuo-spatial consciousness and parieto-occipital areas: a high-resolution EEG study. Cereb. Cortex 16, 37–46 10.1093/cercor/bhi082 15800023

[B6] Bachmann T. (2009). Finding ERP-signatures of target awareness: puzzle persists because of experimental co-variation of the objective and subjective variables. Conscious. Cogn. 18, 804–808 discussion: 809–810. 10.1016/j.concog.2009.02.011 19328726

[B7] Bachmann T. (2011). Attention as a process of selection, perception as a process of representation, and phenomenal experience as the resulting process of perception being modulated by a dedicated consciousness mechanism. Front. Psychol. 2:387 10.3389/fpsyg.2011.00387 22232612 PMC3247680

[B8] Batterink L. Karns C. M. Neville H. (2012). Dissociable mechanisms supporting awareness: the P300 and gamma in a linguistic attentional blink task. Cereb. Cortex 22, 2733–2744 10.1093/cercor/bhr346 22166765 PMC3491763

[B9] Bernat E. Shevrin H. Snodgrass M. (2001). Subliminal visual oddball stimuli evoke a P300 component. Clin. Neurophysiol. 112, 159–171 10.1016/S1388-2457(00)00445-4 11137675

[B10] Block N. (2001). Paradox and cross purposes in recent work on consciousness. Cognition 79, 197–219 10.1016/S0010-0277(00)00129-3 11164028

[B11] Block N. (2005). Two neural correlates of consciousness. Trends Cogn. Sci. 9, 46–52 10.1016/j.tics.2004.12.006 15668096

[B11a] Block N. (2007). Consciousness, accessibility, and the mesh between psychology and neuroscience. Behav. Brain Sci. 30, 481–499 discussion: 499–548. 10.1017/S0140525X07002786 18366828

[B11b] Block N. (2011). Perceptual consciousness overflows cognitive access. Trends Cogn. Sci. 15, 567–575 10.1016/j.tics.2011.11.001 22078929

[B12] Bullmore E. Suckling J. Overmeyer S. Rabe-Hesketh S. Taylor E. Brammer M. (1999). Global, voxel, and cluster tests, by theory and permutation, for a difference between two groups of structural MR images of the brain. IEEE Trans. Med. Imaging 18, 32–42 10.1109/42.750253 10193695

[B13] Cohen M. A. Alvarez G. A. Nakayama K. (2011). Natural-scene perception requires attention. Psychol. Sci. 22, 1165–1172 10.1177/0956797611419168 21841149

[B14] Cohen M. A. Cavanagh P. Chun M. M. Nakayama K. (2012). The attentional requirements of consciousness. Trends Cogn. Sci. 16, 411–417 10.1016/j.tics.2012.06.013 22795561

[B15] Cohen M. A. Dennett D. C. (2011). Consciousness cannot be separated from function. Trends Cogn. Sci. 15, 358–364 10.1016/j.tics.2011.06.008 21807333

[B16] Crick F. Koch C. (1990). Towards a neurobiological theory of consciousness. Semin. Neurosci. 2, 263–275

[B17] Crick F. Koch C. (2003). A framework for consciousness. Nat. Neurosci. 6, 119–126 10.1038/nn0203-119 12555104

[B18] de Graaf T. A. Hsieh P. J. Sack A. T. (2012). The “correlates” in neural correlates of consciousness. Neurosci. Biobehav. Rev. 36, 191–197 10.1016/j.neubiorev.2011.05.012 21651927

[B19] Dehaene S. Changeux J. P. (2011). Experimental and theoretical approaches to conscious processing. Neuron 70, 200–227 10.1016/j.neuron.2011.03.018 21521609

[B20] Dehaene S. Changeux J. P. Naccache L. Sackur J. Sergent C. (2006). Conscious, preconscious, and subliminal processing: a testable taxonomy. Trends Cogn. Sci. 10, 204–211 10.1016/j.tics.2006.03.007 16603406

[B21] Dehaene S. Changeux P. (2004). Neural Mechanisms for Access Consciousness, in The Cognitive Neurosciences, 3rd Edn., ed Gazzaniga M. (Cambridge, MA: MIT Press), 1145–1153

[B22] Dehaene S. Naccache L. (2001). Towards a cognitive neuroscience of consciousness: basic evidence and a workspace framework. Cognition 79, 1–37 10.1016/S0010-0277(00)00123-2 11164022

[B23] Del Cul A. Baillet S. Dehaene S. (2007). Brain dynamics underlying the nonlinear threshold for access to consciousness. PLoS Biol. 5:e260 10.1371/journal.pbio.0050260 17896866 PMC1988856

[B24] Fernandez-Duque D. Grossi G. Thornton I. M. Neville H. J. (2003). Representation of change: separate electrophysiological markers of attention, awareness, and implicit processing. J. Cogn. Neurosci. 15, 491–507 10.1162/089892903321662895 12803962

[B25] Gilchrist A. L. Cowan N. (2010). Conscious and unconscious aspects of working memory, in Unconcious Memory Representations in Perception: Processes and Mechanisms in the Brain, eds Czigler I. Winkler I. (Amsterdam: John Benjamins Publishing Company), 1–36 10.1075/aicr.78.03gil

[B26] Groppe D. Urbach T. Kutas M. (2011). Mass univariate analysis of event-related brain potentials/fields I: a critical tutorial review. Psychophysiology 48, 1711–1725 10.1111/j.1469-8986.2011.01273.x 21895683 PMC4060794

[B27] Harter M. Aine C. (1984). Brain mechanisms of visual selective attention, in Varieties of Attention, eds Parasuraman P. Davis D. (New York, NY: Academic Press), 293–321

[B28] Hassin R. R. Bargh J. A. Engell A. D. McCulloch K. C. (2009). Implicit working memory. Conscious. Cogn. 18, 665–678 10.1016/j.concog.2009.04.003 19442537 PMC2760263

[B29] Hillyard S. A. Anllo-Vento L. (1998). Event-related brain potentials in the study of visual selective attention. Proc. Natl. Acad. Sci. U.S.A. 95, 781–787 10.1073/pnas.95.3.781 9448241 PMC33798

[B30] Koch C. Tsuchiya N. (2007). Attention and consciousness: two distinct brain processes. Trends Cogn. Sci. 11, 16–22 10.1016/j.tics.2006.10.012 17129748

[B31] Koivisto M. Lahteenmaki M. Sorensen T. A. Vangkilde S. Overgaard M. Revonsuo A. (2008). The earliest electrophysiological correlate of visual awareness? Brain Cogn. 66, 91–103 10.1016/j.bandc.2007.05.010 17664036

[B32] Koivisto M. Revonsuo A. (2003). An ERP study of change detection, change blindness, and visual awareness. Psychophysiology 40, 423–429 10.1111/1469-8986.00044 12946115

[B33] Koivisto M. Revonsuo A. (2007). Electrophysiological correlates of visual consciousness and selective attention. Neuroreport 18, 753–756 10.1097/WNR.0b013e3280c143c8 17471060

[B34] Koivisto M. Revonsuo A. (2008). The role of selective attention in visual awareness of stimulus features: electrophysiological studies. Cogn. Affect. Behav. Neurosci. 8, 195–210 10.3758/CABN.8.2.195 18589509

[B35] Koivisto M. Revonsuo A. (2010). Event-related brain potential correlates of visual awareness. Neurosci. Biobehav. Rev. 34, 922–934 10.1016/j.neubiorev.2009.12.002 20005249

[B36] Koivisto M. Revonsuo A. Lehtonen M. (2006). Independence of visual awareness from the scope of attention: an electrophysiological study. Cereb. Cortex 16, 415–424 10.1093/cercor/bhi121 15958780

[B37] Koivisto M. Revonsuo A. Salminen N. (2005). Independence of visual awareness from attention at early processing stages. Neuroreport 16, 817–821 10.1097/00001756-200505310-00008 15891577

[B38] Kouider S. Stahlhut C. Gelskov S. V. Barbosa L. S. Dutat M. de Gardelle V. (2013). A neural marker of perceptual consciousness in infants. Science 340, 376–380 10.1126/science.1232509 23599498

[B39] Lamme V. A. (2006). Towards a true neural stance on consciousness. Trends Cogn. Sci. 10, 494–501 10.1016/j.tics.2006.09.001 16997611

[B40] Lamy D. Salti M. Bar-Haim Y. (2009). Neural correlates of subjective awareness and unconscious processing: an erp study. J. Cogn. Neurosci. 21, 1435–1446 10.1162/jocn.2009.21064 18702582

[B41] Logothetis N. K. Schall J. D. (1989). Neuronal correlates of subjective visual perception. Science 245, 761–763 10.1126/science.2772635 2772635

[B42] Mack A. Clarke J. (2012). Gist perception requires attention. Vis. Cogn. 20, 300–327 10.1080/13506285.2012.666578

[B43] Mack A. Rock I. (1998). Inattentional Blindness. Cambridge, MA: MIT Press

[B44] Marchetti G. (2012). Against the view that consciousness and attention are fully dissociable. Front. Psychol. 3:36 10.3389/fpsyg.2012.00036 22363307 PMC3279725

[B45] Ojanen V. Revonsuo A. Sams M. (2003). Visual awareness of low-contrast stimuli is reflected in event-related brain potentials. Psychophysiology 40, 192–197 10.1111/1469-8986.00021 12820860

[B46] Pins D. ffytche D. (2003). The neural correlates of conscious vision. Cereb. Cortex 13, 461–474 10.1093/cercor/13.5.461 12679293

[B47] Pitts M. A. Martinez A. (2014). Contour integration: sensory, perceptual, and attention-based ERP components, in Cognitive Electrophysiology of Attention: Signals of the Mind, ed Mangun G. R. (San Diego, CA: Academic Press), 178–189 10.1016/B978-0-12-398451-7.00014-2

[B48] Pitts M. A. Martinez A. Hillyard S. A. (2010). When and where is binocular rivalry resolved in the visual cortex? J. Vis. 10, 1–11 10.1167/10.14.25 21191137

[B49] Pitts M. A. Martinez A. Hillyard S. A. (2012). Visual processing of contour patterns under conditions of inattentional blindness. J. Cogn. Neurosci. 24, 287–303 10.1162/jocn_a_00111 21812561

[B50] Pitts M. A. Padwal J. Fennelly D. Martinez A. Hillyard S. A. (2014). Gamma band activity and the P3 reflect post-perceptual processes, not visual awareness. Neuroimage 101, 337–350 10.1016/j.neuroimage.2014.07.024 25063731 PMC4169212

[B51] Railo H. Koivisto M. Revonsuo A. (2011). Tracking the processes behind conscious perception: a review of event-related potential correlates of visual consciousness. Conscious. Cogn. 20, 972–983 10.1016/j.concog.2011.03.019 21482150

[B52] Ramsøy T. Overgaard M. (2004). Introspection and subliminal perception. Phenomenol. Cogn. Sci. 3, 1–23 10.1023/B:PHEN.0000041900.30172.e8

[B53] Sandberg K. Bahrami B. Kanai R. Barnes G. R. Overgaard M. Rees G. (2013). Early visual responses predict conscious face perception within and between subjects during binocular rivalry. J. Cogn. Neurosci. 25, 969–985 10.1162/jocn_a_00353 23281780 PMC4060063

[B54] Schoenfeld M. A. Tempelmann C. Martinez A. Hopf J. M. Sattler C. Heinze H. J. (2003). Dynamics of feature binding during object-selective attention. Proc. Natl. Acad. Sci. U.S.A. 100, 11806–11811 10.1073/pnas.1932820100 12960369 PMC208844

[B55] Sergent C. Baillet S. Dehaene S. (2005). Timing of the brain events underlying access to consciousness during the attentional blink. Nat. Neurosci. 8, 1391–1400 10.1038/nn1549 16158062

[B56] Shafto J. P. Pitts M. A. (2013). Visual processing of faces during inattentional blindness, in Paper presented at the Society for Neuroscience Annual Meeting (San Diego, CA).

[B57] Soto D. Mäntylä T. Silvanto J. (2011). Working memory without consciousness. Curr. Biol. 21, R912–R913 10.1016/j.cub.2011.09.049 22115455

[B57a] Squires K. C. Hillyard S. A. Lindsay P. H. (1973). Vertex potentials evoked during auditory signal detection: relation to decision criteria. Percept. Psychophys. 14, 265–272

[B57b] Squires K. C. Squires N. K. Hillyard S. A. (1975). Vertex evoked potentials in a rating scale detection task: relation to signal probability. Behavioral Biology 13, 21–34 1111506 10.1016/s0091-6773(75)90748-8

[B58] Tallon-Baudry C. (2011). On the neural mechanisms subserving consciousness and attention. Front. Psychol. 2:397 10.3389/fpsyg.2011.00397 22291674 PMC3253412

[B59] Tsuchiya N. Block N. Koch C. (2012). Top-down attention and consciousness: comment on Cohen et al. Trends Cogn. Sci. 16:527 author reply: 528. 10.1016/j.tics.2012.09.004 23026022

[B60] Tsuchiya N. Koch C. (2009). The relationship between consciousness and attention, in The Neurology of Consciousness, eds Laureys S. Tononi G. (San Diego, CA: Elsevier, Ltd). 63–77 10.1016/B978-0-12-374168-4.00006-X

[B61] Tsuchiya N. van Boxtel J. (2013). Introduction to research topic: attention and consciousness in different senses. Front. Psychol. 4:249 10.3389/fpsyg.2013.00249 23641230 PMC3640185

[B62] Vandenbroucke A. R. Fahrenfort J. J. Sligte I. G. Lamme V. A. (2014). Seeing without knowing: neural signatures of perceptual inference in the absence of report. J. Cogn. Neurosci. 26, 955–969 10.1162/jocn_a_00530 24283494

[B63] Verleger R. (2010). Markers of awareness? EEG potentials evoked by faint and masked events, with special reference to the attentional blink, in Unconcious Memory Representations in Perception: Processes and Mechanisms in the Brain, eds Czigler I. Winkler I. (Amsterdam: John Benjamins Publishing Company), 37–70 10.1075/aicr.78.04ver

[B64] Wilenius M. E. Revonsuo A. T. (2007). Timing of the earliest ERP correlate of visual awareness. Psychophysiology 44, 703–710 10.1111/j.1469-8986.2007.00546.x 17584186

[B65] Wilenius-Emet M. Revonsuo A. Ojanen V. (2004). An electrophysiological correlate of human visual awareness. Neurosci. Lett. 354, 38–41 10.1016/j.neulet.2003.09.060 14698477

[B66] Windey B. Vermeiren A. Atas A. Cleeremans A. (2014). The graded and dichotomous nature of visual awareness. Philos. Trans. R. Soc. Lond. B Biol. Sci. 369:20130282 10.1098/rstb.2013.0282 24639587 PMC3965170

[B67] Woodman G. F. Luck S. J. (2003). Dissociations among attention, perception, and awareness during object-substitution masking. Psychol. Sci. 14, 605–611 10.1046/j.0956-7976.2003.psci_1472.x 14629693

[B68] Wyart V. Tallon-Baudry C. (2008). Neural dissociation between visual awareness and spatial attention. J. Neurosci. 28, 2667–2679 10.1523/JNEUROSCI.4748-07.2008 18322110 PMC6671201

[B69] Zinni M. Martinez A. Hillyard S. A. (2014). The neural basis of color binding to an attended object, in Cognitive Electrophysiology of Attention: Signals of the Mind, ed Mangun G. R. (San Diego, CA: Academic Press), 152–164 10.1016/B978-0-12-398451-7.00012-9

