# Supplementary material for: Isolating neural correlates of conscious perception from neural correlates of reporting one's perception
Source: Front Psychol. 2014 Oct 8;5:1078. doi: 10.3389/fpsyg.2014.01078 (PMC4189413; doi:10.3389/fpsyg.2014.01078)
Supplement: Supplementary file 2 [file DataSheet1.PDF]

Supplementary Figures

Figure S1. Results of cluster mass permutation tests for shape minus random difference amplitudes in each of the four main conditions (aware, task-relevant; aware, task-irrelevant; unaware, task-relevant; unaware, task-irrelevant). Separate tests were conducted for the early components (CIN & VAN; 100-300ms) and the late components (LOP & P3b; 300-600ms). Of the 96 total electrodes, 63 electrodes were entered into these analyses (covering central, parietal, temporal, and occipital locations) based on known scalp topographies of the four components of interest. Electrodes on the y-axis are arranged according to spatial position with left hemisphere channels towards the top, midline channels in the middle, and right hemisphere channels towards the bottom. Within each of the three channel groups (L, midline, R), individual channels are arranged from top to bottom according to their anterior-to-posterior (A→P) location on the scalp. Colored squares indicate significant t-values (threshold for display is $p < .01$) at an individual time point for an individual electrode (blue = negative amplitude difference; red = positive amplitude difference), family-wise alpha was $p < .05$. Significant differences from ~160-200ms correspond to the CIN component, ~200-240ms reflect the VAN component, ~300-340ms the LOP component, and ~380-480ms the P3b component.

Figure S1.

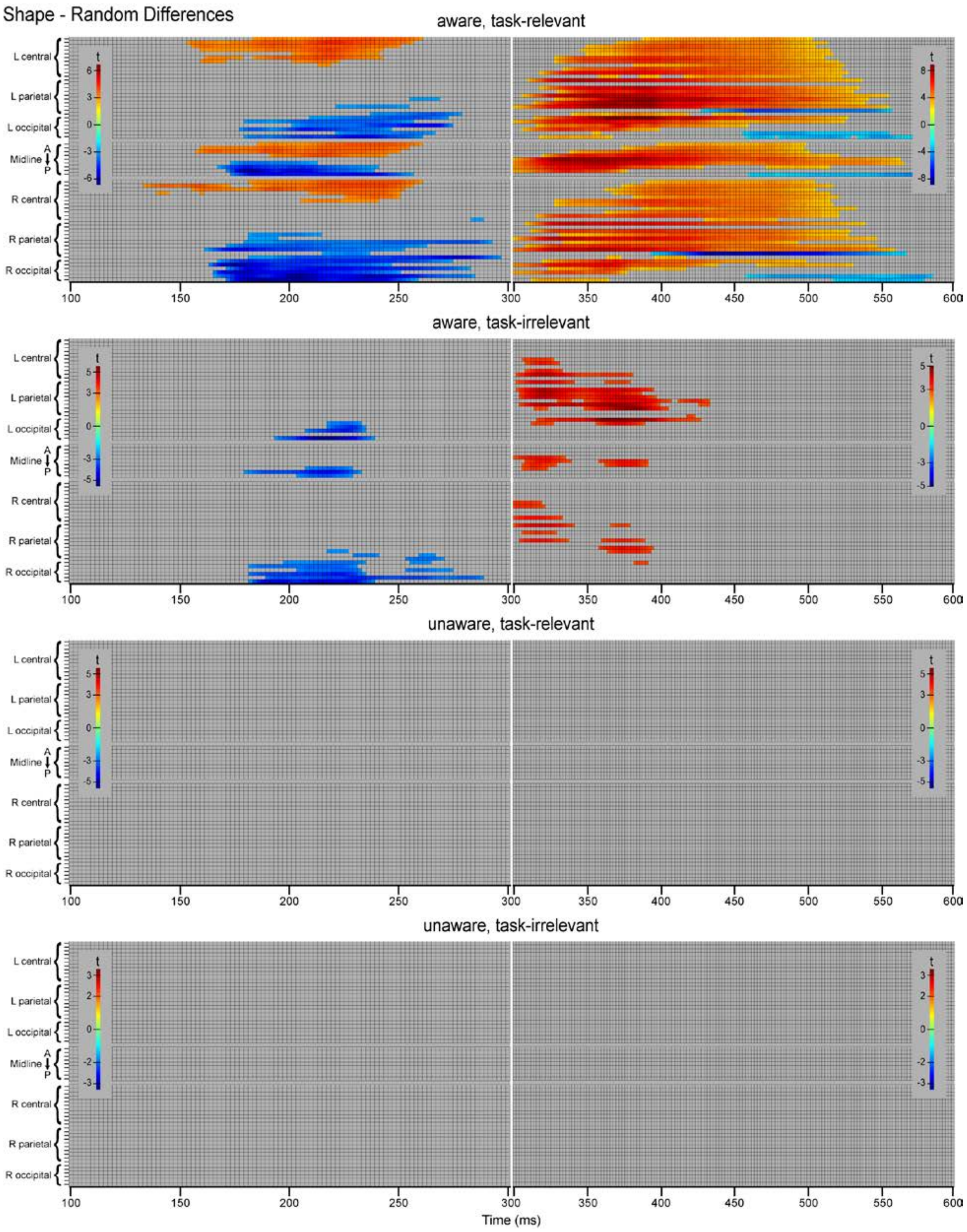

Shape - Random Differences
aware, task-relevant
aware, task-irrelevant
unaware, task-relevant
unaware, task-irrelevant
L central
L parietal
L occipital
Midline
A
P
R central
R parietal
R occipital
t
Time (ms)
100
150
200
250
300
350
400
450
500
550
600

Figure S2. Results of cluster mass permutation tests for color minus random difference amplitudes in each of the four main conditions (aware, task-relevant; aware, task-irrelevant; unaware, task-relevant; unaware, task-irrelevant). Separate tests were conducted for the early components (SEC & VAN; 100-300ms) and the late components (LOP & P3b; 300-600ms). Significant differences from ~130-170ms correspond to the SEC component, ~200-240ms reflect the VAN component, ~290-330ms the LOP component, and ~380-480ms the P3b component.

Figure S2.

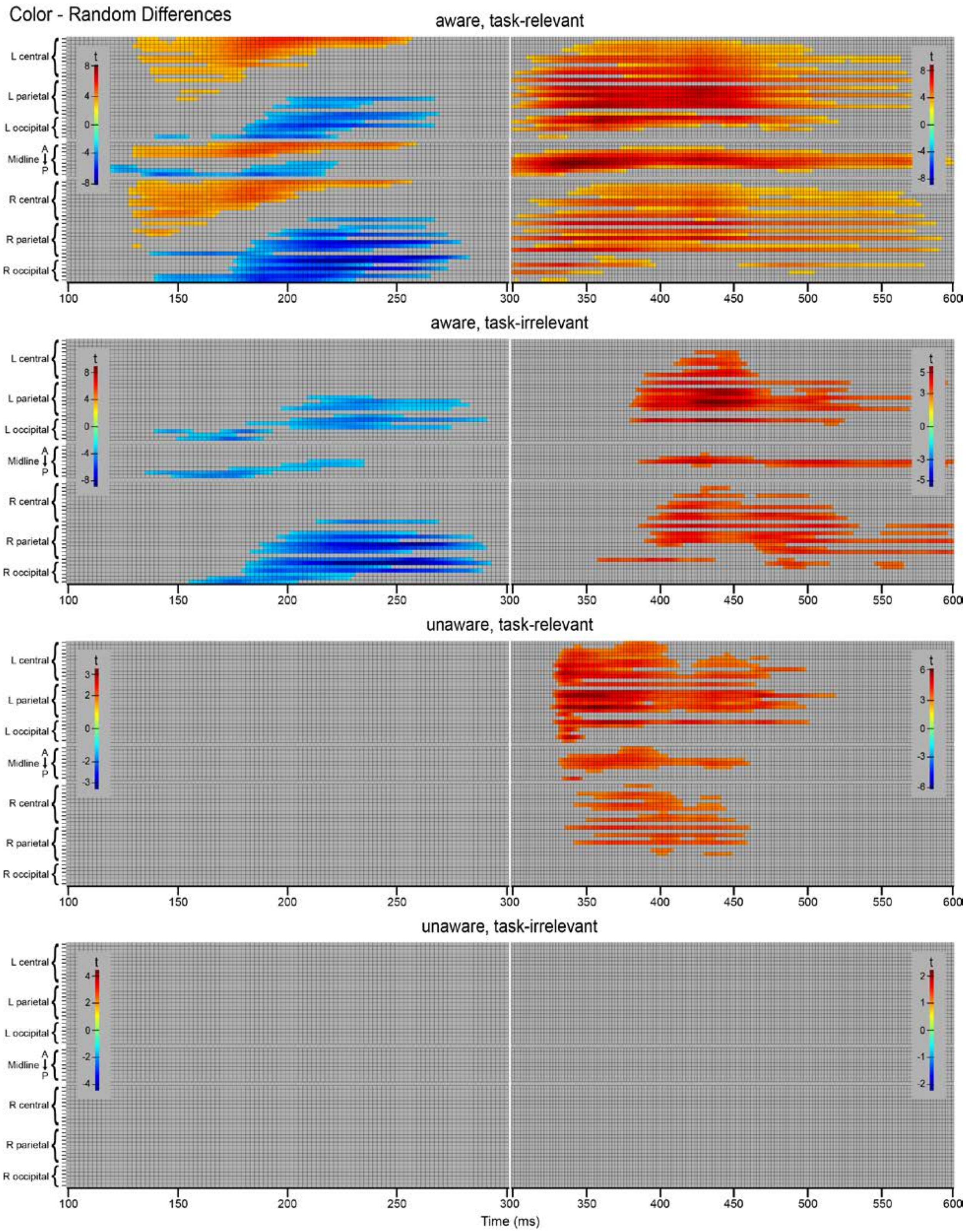
Color - Random Differences
aware, task-relevant
aware, task-irrelevant
unaware, task-relevant
unaware, task-irrelevant
L central
L parietal
L occipital
Midline
A
P
R central
R parietal
R occipital
t
100
150
200
250
300
350
400
450
500
550
600
Time (ms)
